# Design and *in vitro* anticancer assessment of a click chemistry-derived dinuclear copper artificial metallo-nuclease

**DOI:** 10.1093/nar/gkae1250

**Published:** 2025-01-07

**Authors:** Simon Poole, Obed Akwasi Aning, Vickie McKee, Thomas Catley, Aaraby Yoheswaran Nielsen, Helge Thisgaard, Pegah Johansson, Georgia Menounou, Joseph Hennessy, Creina Slator, Alex Gibney, Alice Pyne, Bríonna McGorman, Fredrik Westerlund, Andrew Kellett

**Affiliations:** School of Chemical Sciences, Dublin City University, Glasnevin, Dublin 9, Ireland; Department of Life Sciences, Chalmers University of Technology, Gothenburg, Sweden; School of Chemical Sciences, Dublin City University, Glasnevin, Dublin 9, Ireland; Department of Physics, Chemistry and Pharmacy University of Southern Denmark, Campusvej 55, 5230 Odense M, Denmark; Department of Materials Science and Engineering, University of Sheffield, Sheffield, UK; Department of Nuclear Medicine, Odense University Hospital, Odense, Denmark; Department of Nuclear Medicine, Odense University Hospital, Odense, Denmark; Department of Clinical Research, University of Southern Denmark, Odense, Denmark; Department of Clinical Chemistry, Sahlgrenska University Hospital, Region Vastra Gotaland, Gothenburg, Sweden; Department of Laboratory Medicine, Institute of Biomedicine, Sahlgrenska Academy at University of Gothenburg, Sweden; School of Chemical Sciences, Dublin City University, Glasnevin, Dublin 9, Ireland; School of Chemical Sciences, Dublin City University, Glasnevin, Dublin 9, Ireland; School of Chemical Sciences, Dublin City University, Glasnevin, Dublin 9, Ireland; School of Chemical Sciences, Dublin City University, Glasnevin, Dublin 9, Ireland; Department of Materials Science and Engineering, University of Sheffield, Sheffield, UK; School of Chemical Sciences, Dublin City University, Glasnevin, Dublin 9, Ireland; Department of Life Sciences, Chalmers University of Technology, Gothenburg, Sweden; School of Chemical Sciences, Dublin City University, Glasnevin, Dublin 9, Ireland

## Abstract

Copper compounds with artificial metallo-nuclease (AMN) activity are mechanistically unique compared to established metallodrugs. Here, we describe the development of a new dinuclear copper AMN, Cu_2_-BPL-C6 (BPL-C6 = *bis*-1,10-phenanthroline-carbon-6), prepared using click chemistry that demonstrates site-specific DNA recognition with low micromolar cleavage activity. The BPL-C6 ligand was designed to force two redox-active copper centres—central for enhancing AMN activity—to bind DNA, *via* two phenanthroline ligands separated by an aliphatic linker. DNA-binding experiments, involving circular dichroism spectroscopy, agarose gel electrophoresis and fluorescence quenching, revealed a preference for binding with adenine-thymine-rich DNA. The oxidative cleavage mechanism of Cu_2_-BPL-C6 was then elucidated using *in vitro* molecular and biophysical assays, including in-liquid atomic force microscopy analysis, revealing potent DNA cleavage mediated *via* superoxide and hydrogen peroxide oxidative pathways. Single-molecule analysis with peripheral blood mononuclear cells identified upregulated single-strand DNA lesions in Cu_2_-BPL-C6-treated cells. Using specific base excision repair (BER) enzymes, we showed that Endo IV selectively repairs these lesions indicating that the complex generates apurinic and apyrimidinic adducts. Broad spectrum anticancer evaluation of BPL-C6 was performed by the National Cancer Institute’s 60 human cell line screen (NCI-60) and revealed selectivity for certain melanoma, breast, colon and non-small cell lung cancer cell lines.

## Graphical Abstract

**Graphical Abstract:**
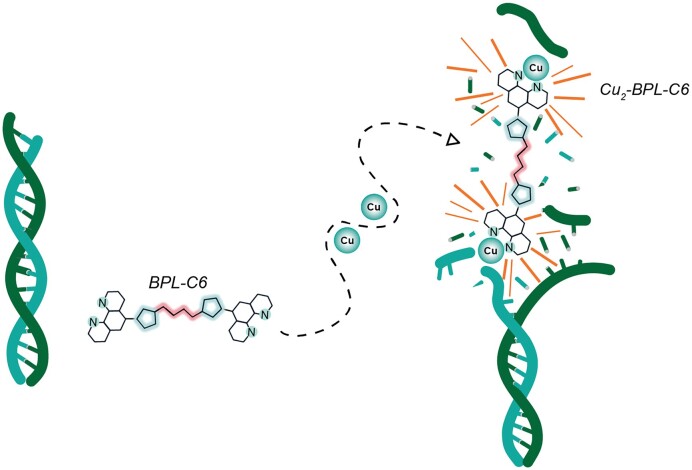

## Introduction

Since its discovery and subsequent structural elucidation, DNA has been a major macromolecular target for manipulation and damage detection (1). This interest has led to the development of a number of synthetic chemical nucleases, such as Sigman’s reagent, which was the first artificial metallo-nuclease (AMN) discovered in 1979 (2,3). AMNs are inorganic complexes with reactive metal centres that mimic the DNA excision machinery of enzymatic nucleases. Their DNA damaging modes can be hydrolytic (4,5) or oxidative, the latter of which occurs through the generation of reactive oxygen species (ROS) associated with metal-oxo species or diffusible radicals that mediate Fenton-type or Haber–Weiss chemistry at the DNA interface (6). DNA damage lesions generated by oxidative AMNs are dissimilar to those produced by classical DNA damaging drugs and are therefore promising new mediators of anticancer activity. Copper complexes are particularly promising as AMNs. Cu-(Phen)_2_ (Phen = 1,10-phenanthroline) is the most well-studied copper-AMN, and it has been shown to oxidatively cleave DNA from the minor groove (6,7). The reduction of Cu(II) to an activated Cu(I) species, and the activation of bioavailable dioxygen, or hydrogen peroxide, results in the generation of the ROS chiefly responsible for abstracting a hydrogen atom from the C1′ position of deoxyribose. The discovery of Cu-(Phen)_2_ has encouraged the development of new chemical nucleases that seek to resolve inherent design limitations including a lack of DNA binding specificity, moderate DNA-binding affinity, dependency on the presence of an exogenous reductant for ROS generation, and facile dissociation of the second coordinated 1,10-phenanthroline ligand that fragments the parent compound.

**Figure 1.**
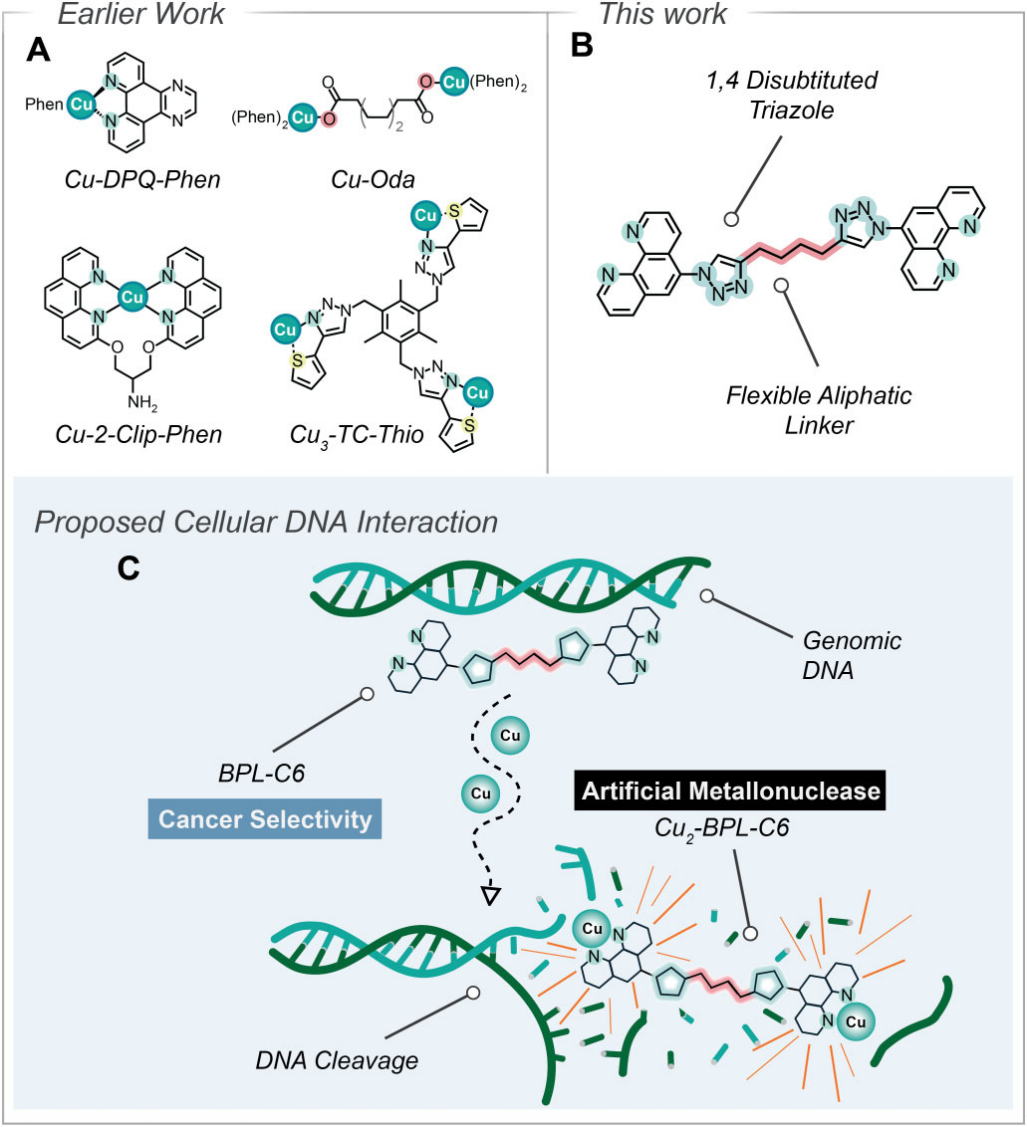
(**A**) The structures of earlier reported AMNs Cu-2-Clip-Phen, Cu-DPQ-Phen, Cu-Oda and Cu_3_-TC-Thio. (**B**) Molecular structure of BPL-C6 prepared using CuAAC click chemistry. (**C**) Upon coordination of two copper ions, Cu_2_-BPL-C6 promotes anticancer activity mediated by AMN activity on genomic DNA.

Attempts to resolve the moderate DNA-binding affinity of Cu-(Phen)_2_ were advanced when Molphy *et al.* reported ternary Cu(II) complexes incorporating DNA intercalators, including Cu-DPQ-Phen (Figure 1A), which were potent DNA binding and oxidative cleavage agents (8). To remediate the high dissociation constant of the second coordinated Phen ligand, Cu-Clip-Phen (Figure 1A) type compounds were developed (9,10). These ligands covalently link two phenanthroline groups with an aliphatic serinol bridge to maintain a 2:1 ligand-to-metal ratio. Furthermore, since the primary amine within the serinol bridge is readily modified, a series of strategies have since emerged whereby directing groups, including minor groove binders (11,12), intercalators (13) and triplex-forming oligonucleotides (14), target chemical nuclease activity toward specific genetic loci. A number of recently reported AMN-active copper complexes present attractive chemotypes for cancer treatment (8,15–18). For example, copper(II) complexes with acyclic caging ligands, including *di*-(2-picolylamine) (19) and *tris*-(2-pyridylmethyl)amine (15), produced promising chemical nuclease activity and were shown to be active against human pancreatic cancer cell lines.

Significant interest has also arisen in discovering polynuclear copper complexes that display self-activated (or unattended) oxidative DNA damage in the absence of co-activating reductants (20,21). One such example is the dinuclear Cu-Oda complex (Figure 1A) (16), which along with potent *in vitro* and intracellular AMN activity, selectively distinguishes TA/TA and AT/AT base pairs and induces a novel ‘Z-like’ DNA binding conformation. One of the key features of Cu-Oda is its ability to mediate DNA double-strand breaks (DSBs) in human cancer cells as validated by γH2AX immunofluorescence (6). Interestingly, the formation of genomic DSBs is influenced by discrete modifications of the copper complex scaffold and follows the general order: di-copper(II) bis-Phen >> mononuclear copper(II) bis-Phen >> mononuclear copper(II) mono-Phen (6). To pursue the role of nuclearity further, a new library of trinuclear copper complexes, called the ‘Tri-Click’ series, were recently developed using copper(I)-catalysed azide-alkyne cycloaddition (CuAAC) click chemistry. This library identified a lead compound with promising chemical nuclease activity, Cu-TC-1 (Supplementary Section S-1 and Supplementary Figure S1) (17), and this series was recently expanded with the discovery of Cu_3_-TC-Thio (18) (Figure 1A) that preferentially binds to the DNA minor groove (18).

Cooperative nucleic acid interactions are not only confined to copper complexes. For example, the *bis*-intercalating Ru-dimer [*μ*-c_4_(cpdppz)_2_(phen)_4_Ru_2_]^4+^ (Supplementary Section S-1 and Supplementary Figure S1), was designed using a tethered *bis*-dppz scaffold with a flexible aliphatic linker in order to enhance DNA binding and to decrease the dissociation rates of the Ru(II) polypyridyl dimer relative to the native and well-studied [Ru(phen)_2_dppz]^2+^ ‘light switch’ complex (Supplementary Section S-1 and Supplementary Figure S1) (22). Another example of cooperative behaviour involves YOYO-1 (Supplementary Section S-1 and Supplementary Figure S1), a *bis*-intercalating dimer of the asymmetric cyanine dye oxazole yellow (YO), which demonstrates high affinity and luminescence upon binding to duplex DNA (23–26).

Herein, we report a new bis-Phen scaffold, BPL-C6 (Figure 1B and C), prepared using the CuAAC click chemistry reaction, that couples two azide-bearing Phen chelators with a terminal dialkyne linker. Motivation for developing BPL-C6 stems from observations that treatment of human cancer cells by polynuclear copper complexes containing bis-Phen ligands leads to an increase in genomic DSBs (6). These intrastrand DNA damage lesions—ostensibly combinations of complex oxidative and bulky adducts—appear as DSB once processed by the cell, particularly during the replication phase. The work seeks to extend the development of earlier AMNs by incorporating two Phen residues into a fixed scaffold—similar to Clip-Phen derivatives—but this motif forces coordination of two redox-active copper centres that facilitate cooperative DNA binding and cleavage affinity. We first describe the synthesis, characterization, biophysical properties of Cu_2_-BPL-C6. Next, the oxidative DNA damaging profile of the complex was explored with supercoiled DNA, and this was followed by a more detailed intracellular examination of these processes using in-liquid atomic force microscopy (AFM) and single-molecule DNA analysis. Additionally, we probed the BPL-C6 ligand for its potential to act as an anticancer prodrug by screening its broad-spectrum cytotoxicity within the National Cancer Institute’s 60 human cell line screen (NCI-60), the results of which were compared to clinically established DNA damaging drugs. Finally, differences in the anticancer activity between the copper(II)-free ligand and the Cu_2_-BPL-C6 metal complex were probed *in vitro* using a number of selected human cancer cell lines.

## Materials and methods

### General remarks

All synthesis was conducted under atmospheric conditions unless otherwise stated. All reagents were purchased from Merck and Tokyo Chemical Industries unless otherwise stated and were used as received. All solvents were obtained commercially and used without further purification. Calf thymus [calf thymus DNA (ctDNA), Ultra-Pure 15 633 019] was purchased from Invitrogen while, poly[d(A-T)_2_] (P0883) and poly[d(G-C)_2_] (P9389) were purchased from Sigma–Aldrich. CutSmart buffer (B7204), pUC19 plasmid (N3041), topoisomerase I (*Escherichia coli*) (M0301) and 100× bovine serum albumin (BSA) (B9000) was purchased from New England Biolabs. Aminoallyl-dUTP-ATTO-647N and YOYO-1 were purchased from Jena Bioscience and Invitrogen, respectively. DNA hairpins with AlexaFluor647 and IOWA black quencher modification were custom designed and purchased from Integrated DNA Technologies. Prodigiosin was purchased from MedChemExpress (HY-100711).

### NMR

NMR experiments were conducted at room temperature using either CDCl_3_ or deuterated dimethyl sulfoxide (DMSO-d_6_) obtained from Sigma–Aldrich. Solvents were used without further purification. All ^1^H-NMR were obtained on a Bruker Avance Ultrashield 600 MHz instrument. Intermediates were analysed by ^1^H-NMR and the final ligand was characterized by ^1^H-NMR and ^13^C-NMR. All ^1^H-NMR, and ^13^C-NMR spectra are contained in supplementary information (Supplementary Section S-2 and Supplementary Figures S2–S6). Data was processed and analysed using Mnova software (v15.0.1).

### Fourier-transform infrared spectroscopy

All Fourier-transform infrared spectroscopy data were collected at room temperature using a Perkin Elmer Spectrum Two ATR Spectrometer (Supplemental Section S-3 and Supplementary Figures S7–S10). Data was processed and analysed using GraphPad Prism (V10.1.1).

### Mass spectrometry

Electrospray-ionization mass spectrometry (ESI-MS) analyses were conducted using a MaXis HD quadrupole ESI-QTOF mass spectrometer. A solution of 10 μL of BPL-C6 (10 mM) was taken and mixed with various equivalents of copper(II) nitrate trihydrate and diluted to a final volume of 500 μL in H_2_O, yielding 200 μM of each sample, generating a series of stoichiometric Cu(II):BPL-C6 solutions between 0:1 and 10:1 (Supplementary Section S-4, and Supplementary Figures S11–S13). Analyses were performed in ESI positive mode with the capillary voltage was set to 6000 V, nebulizing gas at 0.8 bar, and drying gas at 2 L/min at 180°C. The TOF scan range was from 50 to 2500 mass-to-charge ratio (*m/z*). ESI-MS of BPL-C6 and the discrete Cu_2_-BPL-C6 complex were conducted under the same conditions (Supplementary Section S-4, and Supplementary Figures S14 and S15). Data processing was performed using the Compass Data Analysis software version 4.3 (Bruker Daltonik GmbH, Bremen, Germany) and ChemDraw software (V20.0).

### Crystallography

Single crystal X-ray diffraction data were collected on a Synergy, Dualflex, AtlasS2 diffractometer. Crystal Data for C_34_H_26_N_10_Cl_6_ (*M* = 787.35 g/mol): monoclinic, space group *P*2_1_/_n_ (no. 14), *a* = 6.9059(14) Å, *b* = 15.297(3) Å, *c* = 16.276(3) Å, *β* = 98.35(2)°, *V* = 1701.1(6) Å^3^, *Z* = 2, *T* = 100.00(10) K, μ(Cu Kα) = 4.967 mm^-1^, *D_calc_* = 1.537 g/cm^3^, 3329 reflections measured (7.972° ≤ 2θ ≤ 101.968°), 1769 unique (*R_int_* = 0.0820, *R_sigma_* = 0.1401) which were used in all calculations. The final *R_1_* was 0.1098 (I > 2σ(I)) and *wR_2_* was 0.3246 (all data). For refinement details, see supplementary data section (Supplementary Section S-5, Supplementary Figures S16–S20 and Supplementary Table S1).

### Elemental analysis

Samples were analysed using a Flash EA Elemental Analyser and Eager 300 software. Two to four milligrams of sample was placed in a tin capsule and pyrolyzed at a temperature >1000°C with a portion of O_2_ gas. The resultant gases were passed through a quartz reactor tube containing copper/chromium oxide and silvered cobaltous oxide where they are purified and reduced. The emerging gases were separated on a PTFE Multiseparation Column and detected using a thermal conductivity detector. The percentage of carbon, nitrogen and hydrogen was determined from the resulting chromatographic peaks. The carrier gas used was helium at a rate of 130 mL/min and a run time of 530 s. The instrument was calibrated before use against a certified nicotinamide standard (Supplementary Section S-6 and Supplementary Figure S21).

### Synthesis of BPL-C6

#### 5,6-Epoxy-5,6-dihydro-1,10-phenanthroline

5,6-Epoxy-5,6-dihydro-1,10-phenanthroline, **1**, was synthesized according to previously published literature with modifications (27). 1,10-phenanthroline.H_2_O (1.8056 g, 9.11 mmol, 1 equivalent) was added to a biphasic mixture of 170 mL CHCl_3_ and 150 mL H_2_O with stirring. Separately, NaOCl.5H_2_O (44.4280 g, 270.06 mmol) and tetrabutylammonium bisulphate (1.7183 g, 5.06 mmol) were added to a conical flask with 150 mL H_2_O and sonicated until full dissolution was achieved. This mixture was added to the 1,10-phenanthroline solution. Subsequently, the entire mixture was vigorously stirred for 2 h at room temperature (r.t.) The organic layer was separated while the aqueous layer was washed with 2× 100 mL portions CHCl_3_. These aqueous layer washings were combined with the initial organic fraction, dried with Na_2_SO_4_ and evaporated to obtain a crude yellow-brown oil. The oil was dissolved in CHCl_3_, further reduced under vacuum prior to final drying overnight under vacuum. The oil was purified by addition of 2 mL *t*-butyl alcohol and sonicated to obtain a beige precipitate. The precipitate was washed successively with additional portions of *t*-butyl alcohol and finally dried over vacuum to afford the pure product as an off-white powder. Yield: 0.7612 g, 43%; ^1^H NMR (600 MHz, CDCl_3_) δ 8.94 (dd, *J* = 4.7, 1.7 Hz, 2H), 8.05 (dd, *J* = 7.6, 1.7 Hz, 2H), 7.44 (dd, *J* = 7.6, 4.7 Hz, 2H), 4.66 (s, 2H). IR (ATR): 3655.2, 2983.8, 2888.4, 1558.57, 1427.8, 881.61, 804.55, 749.19, 703.01, 613.11, 540.27 cm^−1^.

#### 5-Azido-1,10-phenanthroline

5-Azido-1,10-phenanthroine, **2**, was synthesized in accordance with previously published literature (28). Yield: 0.1265 g, 57%; ^1^H NMR (600 MHz, CDCl_3_) δ 9.24 (dd, *J* = 4.3, 1.7 Hz, 1H), 9.13 (dd, *J* = 4.3, 1.7 Hz, 1H), 8.54 (dd, *J* = 8.3, 1.7 Hz, 1H), 8.19 (dd, 1H), 7.69–7.62 (m, 2H), 7.48 (s, 1H). IR (ATR): 2110.50, 1419.30, 1316.23, 1268.16, 1001.00, 831.22, 799.66 740.07, 623.52, 534.76 cm^−1^.

#### BPL-C6 (1, 4-Bis(1-(1,10-phenanthrolin-5-yl)-1H-1, 2, 3-triazol-4-yl)butane)

5-Azido-1,10-phenanthroline (0.2035 g, 0.92 mmol) was dissolved in 10 mL EtOH and heated to 85 °C under inert conditions. Separately, CuBr (0.1302 g, 0.91 mmol) was dissolved in 5 mL MeCN and diisopropylethylamine (DIPEA) (157.48 μL, 0.90 mmol) was added; 1,7-octadiyne (0.0449 g, 0.42 mmol) was dissolved in 15 mL of EtOH. The green CuBr mixture was quickly added to the 1,7-octadiyne solution which turned bright yellow. This mixture was added to the 5-azido-1,10-phenanthroline solution and stirred for 16 h in darkness at 85°C. The mixture was cooled and the volume reduced under vacuum. H_2_O (10 mL) was added, and the mixture was sonicated prior to the addition of 0.5 M ethylenediaminetetraacetic acid (EDTA) (30 mL) with stirring for 2 h. The aqueous layer was extracted with CHCl_3_ (4 × 100 mL). The organic layer was washed with H_2_O (3 × 25 mL) and brine (25 mL), dried with Na_2_SO_4_ and reduced under vacuum to obtain a crude beige-orange oil. The product was obtained by precipitation with a cold MeOH solution and sonication. Finally, the purified product was afforded by filtration, washed with excess cold MeOH and Et_2_O and collected as a light beige powder, **3**. The compound was crystallized using slow vapour diffusion and verified using single crystal X-ray diffraction (XRD) (Supplementary Section S-5 and Supplementary Figures S16–S20). Yield: 0.1314 g, 57%; ^1^H NMR (600 MHz, CDCl_3_) δ 9.29 (ddd, *J* = 8.9, 4.3, 1.7 Hz, 4H), 8.32 (dd, *J* = 8.1, 1.8 Hz, 2H), 8.19 (dd, *J* = 8.4, 1.7 Hz, 2H), 7.98 (s, 2H), 7.84 (s, *J* = 0.7 Hz, 2H), 7.74 (dd, *J* = 8.1, 4.3 Hz, 2H), 7.70 (dd, *J* = 8.4, 4.3 Hz, 2H), 3.02 (q, *J* = 5.1 Hz, 4H), 2.04 (p, *J* = 3.8 Hz, 4H). ^13^C NMR (151 MHz, CDCl_3_) δ 151.71, 151.27, 148.22, 146.39, 146.10, 136.47, 132.11, 131.73, 126.90, 124.54, 123.89, 123.76, 123.44, 123.32, 28.84, 25.28. ESI-MS: [M + Na]^+^*m/z* calculated; 571.21 *m/z*; found = 571.2078; [M + H]^+^*m/z* calculated; 549.23 *m/z*; found = 549.23 IR (ATR): 3051.5, 2935.93, 2858.83, 1628.70, 1587.60, 1496.65, 1465.63, 1420.95, 1344.56, 1223.46, 1151.43, 1128.24, 1102.87, 1079.10, 1035.33, 1012.64, 927.59, 888.13, 870.81, 798.95, 742.67, 657.71, 650.33, 623.81, 596.10, 514.37 cm^−1^.

### Complex preparation

#### Cu_2_-BPL-C6 [Cu_2_(BPL-C6)(NO_3_)_4_.3H_2_O]

Cu(NO_3_)_2_.3H_2_O (44 mg, 0.1823 mmol) was added to 5 mL EtOH in a 50 mL two-neck round bottom flask at 70°C. Separately, BPL-C6 (50 mg, 0.0911 mmol) was added to a large vial containing 5 mL EtOH. The BPL-C6 suspension was solubilized in the ethanolic solution at 50°C and transferred to the stirring solution of Cu(NO_3_)_2_.3H_2_O dropwise. A further 5 mL of EtOH was used to ensure all of the BPL-C6 solution was transferred, and the reaction was allowed to reflux at 90°C. The mixture was allowed to cool to room temperature and a green/blue precipitate, **4**, appeared when the reaction was complete. The precipitate was collected under vacuum and washed with excess EtOH, H_2_O and diethyl ether. Yield: 0.0753 g (0.0814 mmol, 89%), IR (ATR): 3137.70, 3067.70, 2943.41, 1643.82, 1593.52, 1527.45, 1475.62, 1425.00, 1284.44, 1155.34, 1166.63, 1081.83, 1040.80, 1003.33, 949.52, 893.14, 826.78, 730.48, 650.33. ESI-MS: [(C_32_H_24_)Cu_2_(NO_3_)_3_]^+^*m/z* calculated; 860.04 *m/z*; found = 860.04. Elemental analysis calculated (%) for (C_32_H_24_N_10_)Cu_2_(NO_3_)_4_.3H_2_O: C 39.31, H 3.09, N 20.06; found C 39.20, H 2.85, N 20.24.

## DNA-binding experiments

### Solution preparation

BPL-C6 was initially prepared in dimethylformamide (DMF) or DMSO and further diluted in water. The metal complex of BPL-C6, designated Cu_2_-BPL-C6, was prepared in-situ by co-incubating the ligand with two equivalents of copper(II) nitrate trihydrate for 30 min at 37°C prior to DNA-binding analysis.

### Competitive ethidium bromide displacement assay

A preliminary screen of metal ions including Cu(II), Zn(II), Mn(II), Ni(II), Co(II) and Fe(II) co-incubated with BPL-C6 was analysed using a single point experiment to compare their binding efficacy with ctDNA (Supplementary Section S-7 and Supplementary Figure S22). The DNA-binding affinity of Cu_2_-BPL-C6 was determined as the lead agent and was subsequently analysed using a 1 h incubation period with working solutions of DNA {20 μM ctDNA and synthetic alternating co-polymers poly[d(A-T)_2_)] and poly[d(G-C)_2_]} by ethidium bromide (EtBr) (25.2 μM working solution) fluorescence quenching to afford a final concentration of 12.5 μM EtBr, 12.5 μM DNA in 100 μL, in a similar manner to the high throughput method previously reported by Kellett *et al.* (29). The influence of Cu_2_-BPL-C6 on native EtBr fluorescence was investigated to ensure no interference with EtBr fluorescence was observed under these conditions (Supplementary Section S-7 and Supplementary Figure S23). A subsequent copper(II) dependency study was conducted whereby BPL-C6 was treated with varying ratios of copper(II) nitrate 0:1–8:1 [Cu(II):BPL-C6]. Thereafter, ctDNA was treated with Cu_2_-BPL-C6 at varying concentration. In a similar manner, poly[d(A-T)_2_)] and poly[d(G-C)_2_] were treated with Cu(II):BPL-C6 2:1 post EtBr saturation. The apparent binding constant was determined spectrophotometrically by monitoring the fluorescence of EtBr using a Tecan Spark Multimode plate reader. Each drug concentration was measured in triplicate, and the apparent binding constants were calculated using *K*_app_ = *K*_b_× 12.6/C_50_ where *K*_b_ = 8.8 × 10^6^ M^−1^. The data was plotted and analysed using GraphPad Prism (v10.1.1). Excitation/emission wavelengths for EtBr detection were 530/590 nm.

### Limited-bound fluorescence quenching

Fluorescence quenching assays were conducted as reported by Molphy *et al.* with slight modification (8). Solutions of alternating copolymers poly[d(A-T)_2_] and poly[d(G-C)_2_] were prepared using nuclease free water and quantified on a NanoDrop One (ThermoFisher) using A_260_ and extinction coefficients of 13,100 (bp)^-1^ cm^−1^ and 16,800 (bp)^-1^ cm^−1^, respectively; 2× working solutions were prepared containing 10 μM EtBr and 50 μM of poly[d(A-T)_2_] or poly[d(G-C)_2_] in 80 mM HEPES (2-[4-(2-hydroxyethyl)-1-piperazinyl]-ethanesulfonic acid), 40 mM NaCl at pH 7.2; 2× ‘blank’ solutions were prepared using the same conditions, but the DNA copolymer was excluded. A serial dilution of Cu_2_-BPL-C6 was performed in triplicate on a 96-well plate such that all sample wells had a total volume of 50 μL, which contained 80 mM HEPES, 40 mM NaCl and varying concentrations of Cu_2_-BPL-C6. 50 μL of either the poly[d(A-T)_2_] or poly[d(G-C)_2_] working solution was then added to each experimental well. Control wells (no Cu_2_-BPL-C6) contained 50 μL of 2× working solution and 50 μL of buffer. Blank wells (no Cu_2_-BPL-C6 or DNA copolymer) contained 50 μL of 2× ‘blank’ solution and 50 μL of buffer. DNA-binding affinity of Cu_2_-BPL-C6 with ctDNA was conducted using a 1 h incubation period using the same conditions (Supplementary Section S-7 and Supplementary Figure S24). EtBr fluorescence was measured on a Tecan Spark Multimode plate reader with excitation and emission at 530 and 590 nm, respectively, and a bandwidth of 5 nm. Fluorescence was normalized using Eq 1 and fit using nonlinear regression in GraphPad Prism (v10.1.1).


(1) \begin{equation*}{\rm Fractional}\ {\rm Fluoresence} = \ \frac{{{{F}_{{\rm test}}} - \ {{F}_{{\rm blank}}}}}{{{{F}_{{\rm control}}} - \ {{F}_{{\rm blank}}}}}\end{equation*}

### Viscosity

The viscosity (*η*) of ctDNA was monitored in solution upon increasing addition of Cu_2_-BPL-C6. Changes in viscosity compared to the native DNA solution (*η – η_0_*) were calculated according to the method earlier reported by this lab (29). Using a DV-II-Programmable Digital Viscometer equipped with Enhanced Brookfield UL Adapter, viscosity was monitored at room temperature by gradually increasing the [complex]/[DNA] ratio (*r-*value) between 0.02 and 0.20 (30).

### Circular dichroism spectroscopy

Cu_2_-BPL-C6–DNA interactions were analysed within Starna quartz cuvettes using an Applied Photophysics Chirascan-Plus circular dichroism (CD) spectrometer. Solutions of oligonucleotides containing ctDNA (*ε*_260_ = 12,824 M (bp)^−1^ cm^−1^), poly[(d(A-T)_2_] (*ε*_260_ = 13,100 M (bp)^−1^ cm^−1^), and poly[(d(G-C)_2_] (*ε*_260_ = 16,800 M (bp)^−1^ cm^−1^) were melted and annealed prior to quantification by measuring absorbance at 260 nm using an Agilent Cary 100 dual beam spectrophotometer and a Nanodrop 1000 (Themo Fisher Scientific) to give 50 μM working solutions in 10 mM NaCl. DNA solutions were incubated with Cu_2_-BPL-C6 using an *r*-value between 0.1 and 0.2 (where *r* = [drug]/[DNA]) for 10 min periods at 37 °C for ctDNA, poly[d(A-T)_2_] and poly[d(G-C)_2_] DNA. Spectra were captured in the range of 190–350 nm at 37°C with 1.0 nm increments, a time point of 1 s and a bandwidth of 1.0 nm.

### Fluorescence melting

Thermal melting analysis was performed according to Gibney *et al.* with modifications (18). Pre-annealed FRET labelled hairpin DNA (fluorophore = AlexaFluor647, quencher = IOWA black) was added to each sample for analysis and prepared at a final concentration of 1.0 μM DNA and Cu_2_-BPL-C6 with *r* values of 0–10 (0.5 increments, *r* = [drug]/[DNA]), incubated at 37°C for 30 min (with the exception of FRET-1, where the max *r* loading was *r*= 7 due to DNA condensation effects). Fluorescence melting was performed in triplicate on a LightCycler 480 II (Roche) at a ramp rate of 0.5 °C per minute up to a maximum of 95 °C. T_m_ values were taken as the half-maximal inhibitory concentration (IC_50_) of the normalized melting curves. Fraction bound analysis was next plotted using the maximum ratio of drug:DNA as the fully bound state, and fit with the Bard equation to provide binding affinity and binding site size information for each hairpin (FRET-1, *ε*_260_ = 358,006 M (bp)^−1^ cm^−1^), (FRET-2, *ε*_260_ = 375,406 M (bp)^−1^ cm^−1^), (FRET-3, *ε*_260_ = 361,206 M (bp)^−1^ cm^−1^) and (FRET-4, *ε*_260_ = 307,100 M (bp)^−1^ cm^−1^). Data was processed and analysed using GraphPad Prism (V10.1.1). Hairpin DNA sequences employed for fluorescence melting experiments:

FRET-1 5′-F-CGCGAATTCGCGAAAAACGCGAATTCGCG-Q-3′

FRET-2 5′-F-GCATTATAATGCAAAAAGCATTATAATGC-Q-3′

FRET-3 5′-F-ATCGGCGCCGATAAAAAATCGGCGCCGAT-Q-3′

FRET-4 5′-F-ATGGCCGGCCGGAAAAACCGGCCGGCCAT-Q-3′

F = AlexaFluor647; Q = IOWA black

### Microscale thermophoresis

The DNA-binding affinity of Cu_2_-BPL-C6 to 5′-AlexaFluor647 fluorescently labelled DNA hairpin was determined using a Monolith instrument (Nanotemper Technologies GmbH) in a similar manner to methods previously reported (18,31). Cu_2_-BPL-C6 was diluted from a 2.0 mM master stock to 200 μM in 80 mM HEPES buffer and 25 mM NaCl. The buffer (15 μL) was added to samples 1–16. Cu_2_-BPL-C6 (200 μM, 30 μL) was added to sample 16 with a 2:1 dilution factor (drug:DNA) to afford a final maximum concentration of 125 μM. A serial dilution was performed by mixing 30 μL of sample 16 with the 15 μL of buffer in sample 15, and so. Pre-annealed fluorescently labelled hairpin DNA (2 μM, 15 μL) was then added to all samples and mixed to afford a final DNA concentration of 1 μM. Samples were centrifuged to remove bubbles and were then immediately loaded into a glass capillary (Monolith standard capillary, NanoTemper Technologies GmbH, MO-K022) and placed into the Monolith sample tray. Care was taken to avoid touching the centre of the capillary. Microscale thermophoresis (MST) binding affinity was performed with MST power set to high and excitation power set to 1% in the red channel. MST measurements were repeated in triplicate, and data was plotted in GraphPad Prism (10.1.1) (F-1, *ε*_260_ = 313,500 M (bp)^−1^ cm^−1^), (F-2, *ε*_260_ = 330,900 M (bp)^−1^ cm^−1^), (F-3, *ε*_260_ = 316,700 M (bp)^−1^ cm^−1^) and (F-4, *ε*_260_ = 307 100 M (bp)^−1^ cm^−1^). Hairpin DNA sequences employed for MST experiments are as follows:

F-1 5′-F-CGCGAATTCGCGAAAAACGCGAATTCGCG-3′

F-2 5′-F-GCATTATAATGCAAAAAGCATTATAATGC-3′

F-3 5′-F-ATCGGCGCCGATAAAAAATCGGCGCCGAT-3′

F-4 5′-F-ATGGCCGGCCGGAAAAACCGGCCGGCCAT-3′

F = AlexaFluor647

### DNA nuclease and ROS scavenger studies

Procedures were adapted from previously published protocols (8,32), where reactions were carried out in 80 mM HEPES (pH 7.2) unless otherwise stated and followed the general procedure: Assays were performed in 20 μL of 80 mM HEPES buffer (pH 7.2) with 25 mM of NaCl, 1 mM of Na-*L*-ascorbate, 400 ng of superhelical pUC19 plasmid DNA (NEB, N3041) and increasing concentrations of each tested complex. Samples were incubated at 37 °C for 30 min. Quenching was performed using 6× DNA loading dye (ThermoScientific, R0611) containing 10 mM Tris-HCl, 0.03% bromophenol blue, 0.03% xylene cyanole FF, 60% glycerol, 60 mM EDTA, and was added to each sample and loaded onto an agarose gel (1.3%) containing 10 μL SYBR Safe. Electrophoresis was carried out at 70 V for 1.5 h in 1× TAE (tris-acetate-EDTA) buffer. Self-activation gels were performed using the same conditions in the absence of Na-*L*-ascorbate. Radical scavenger assays were performed were treated the same in the presence of 1.0 μL of 10 mM ROS scavenger stocks: dimethylthiourea (DMTU) (H_2_O_2_, 10 mM); NaN_3_ (^1^O_2_, 10 mM); tiron (O_2_^•−^, 10 mM); *D*-mannitol (^•^OH, 10 mM); *L*-methionine (H_2_O_2_, ^•^OH, HOCl, 10 mM); and *L*-histidine (^1^O_2_, 10 mM). Gels were captured and analysed using a UV transilluminator (G:Box mini 9, GeneSys software, Syngene).

### Topoisomerase inhibition

The topoisomerase I relaxation assay was carried out using a previously reported method with slight modification (33). Experiments were carried out with 400 ng of plasmid pUC19 DNA, followed by addition of varying concentrations of Cu_2_-BPL-C6 (0.1–50 μM) complex. Each reaction was pre-incubated in darkness for 30 min at room temperature and the final reaction volume was 20 μL using 80 mM HEPES buffer (pH 7.2), and CutSmart^®^ buffer. Next, topoisomerase I enzyme (1 unit) was added to all samples with exception of the control DNA and the mixtures were incubated for 20 min at 37°C in darkness. To quench the enzymatic reaction sodium dodecyl sulfate (0.25%) and protein kinase (250 μg/mL) were added, and the samples were incubated for 30 min at 50°C. The reactions were quenched by adding 6× Fermentas loading buffer containing 10 mM Tris-HCl, 0.03% bromophenol blue, 0.03% xylene cyanole FF, 60% glycerol and 60 mM EDTA. The samples were then loaded onto 1.25% native agarose gel in 1× Tris-borate-EDTA (TBE) buffer and electrophoresis was performed at 60 V for 2.5 h in 1× TBE buffer. The gel was then visualized by staining with SYBR™ safe (20 μL of dye in 100 mL H_2_O) for 30 min, followed by soaking in a water bath for 15 min, and then imaged using a UV transilluminator (G:Box mini 9, GeneSys software, Syngene).

### Atomic force microscopy

#### Sample preparation

For Cu_2_-BPL-C6 treated samples in the absence of *L*-ascorbate, 2686 bp pUC19 plasmid (New England Biolabs) was suspended in buffer solution (80 mM HEPES, 25 mM NaCl) to a final concentration of 20 ng/μL. Cu_2_-BPL-C6 (or Cu-Prodigiosin) was then added to a final concentration of 20 μM. The sample was incubated at 37°C and sampled at the selected time points. At the 180 min time point, 200 μM EDTA was added and incubated for 15 min. For the samples in the presence of *L*-ascorbate, the plasmids were buffered as before, with the addition of 1 mM of *L*-ascorbate. In this case Cu_2_-BPL-C6 was added at a concentration of either 10 or 15 μM and incubated at 37°C for 30 min. For each sample, 1 μL was removed and diluted in 2 μL of milliQ water in preparation for immobilization (final DNA concentration of 6.7 ng/μL). From this dilution 1 μL was immobilized on a freshly cleaved mica disk in 20 μL of immobilization buffer (25 mM MgCl_2_, 10 mM TRIS, pH 7.4) for 5 min. The mica was then washed four times with 20 μL imaging buffer (3 mM NiCl_2_, 20 mM HEPES, pH 7.4), and a further 20 μL was added for imaging.

#### Imaging

All AFM measurements were performed in liquid following a previously published protocol (34). All experiments were carried out in PeakForce Tapping imaging mode on a FastScan Dimension XR AFM system (Bruker), using FastScan D (Bruker) probes. The PeakForce amplitude was set to 5 nm, the PeakForce Tapping frequency to 12 kHz and the PeakForce setpoints in the range: 7–15 mV, corresponding to peak forces of <70 pN. Large area scans (2 × 2 μm) were recorded at 1024 × 1024 pixels at line rates of ∼3–5 Hz.

#### Image processing

The freely available, open-source software TopoStats (35) was used to process raw AFM data and analyse the DNA molecules (36), which is configured using a file that can be found with the dataset (37). Briefly, the software loaded raw AFM images, carried out flattening, both line-by-line and plane flattening. Individual molecules were masked based on a height threshold to separate them from the background. A second flattening was carried out which excluded the grain, improving the flattening of the image. The height distribution of the flattened image was then shifted vertically to set the background to zero by calculating the mean of the non-grain containing data and subtracting that value from the image. Finally, a 1.1 px Gaussian filter was applied to reduce any high-gain noise. Statistics on the grains were collated from ‘allstatistics.csv’. Categorization of the pUC19 molecules as either ‘Circular’ or ‘Linear’ was performed by manual counting. The total volume of the grains was used to determine condensation of multiple molecules. Aggregates were considered as ‘large’ when >5 × 10^4^ nm^3^ in volume. The ‘Smallest Bounding Area’ of the molecule is defined as the area of the smallest bounding box which can be placed around each circular molecule. This was only performed on low volume grains (<80 000 nm^3^) to probe the area of individual DNA molecules.

### Single-molecule DNA damaging assay

#### Chemicals

The protocol was adapted from Singh *et al.* (38). *D*-Mannitol, *L*-histidine, *L*-methionine, tiron, β-mercaptoethanol (BME), GenElute-Mammalian Genomic DNA miniprep kit and YOYO-1 were purchased from Invitrogen. Allyltrimethoxysilane (ATMS) and (3-aminopropyl)triethoxysilane (APTES) were purchased from Sigma–Aldrich. CutSmart buffer, NEBuffer 2, deoxynucleotide (dNTP) Solution Set (N0446S) and repair enzymes – Endo III (M0268S), Endo IV (M0304S), Endo VIII (M0299S), hAAG (M0313S), APE1 (M0282S), Fpg (M0240S) and UDG (M0280S) were purchased from New England Biolabs. DNA polymerase I (M2055) and aminoallyl-dUTP-ATTO-647N were purchased from Promega and Jena Bioscience respectively. Glass coverslips and microscope slides were purchased from Thermo Fischer Scientific and VWR, respectively.

#### Blood sample collection

Excess blood (EDTA tubes) from individuals with normal differential blood count was collected from the Hematology Lab (Clinical Chemistry Department) at Sahlgrenska University Hospital in Gothenburg, Sweden. Peripheral mononuclear blood cells (PBMCs) were isolated from the blood by density gradient centrifugation using Lymphoprep (Axis-Shield PoC AS, Oslo, Norway) according to the manufacturer’s instructions and resuspended in RPMI 1640 media prior to treatment.

#### Treatment of PBMCs with Cu_2_-BPL-C6

10 mM and 100 mM stock solutions of BPL-C6 and copper(II) nitrate trihydrate were prepared in DMSO and Milli Q^®^ (MQ) water. PBMCs were treated with Cu_2_-BPL-C6 (150 μM) for 1 h at 37°C.

#### Treatment of PBMCs with antioxidants

100 mM stock solutions of *D*-mannitol, *L*-histidine, *L*-methionine and tiron were prepared in MQ water. PBMCs were pre-treated with 1 mM of each scavenger for 2 h prior to treatment with Cu_2_-BPL-C6 (150 μM) for 1 h at 37°C.

#### Extraction of DNA

Drug and antioxidant treatment of PBMCs were followed by addition of proteinase K and DNA extraction using GenElute-Mammalian Genomic DNA Miniprep Kit following manufacturer’s instructions. DNA concentrations were measured using a NanoDrop 1000 spectrophotometer. Shear-induced fragmentation of the DNA was minimized by using wide bore pipette tips throughout the procedure.

#### Fluorescent labelling of DNA damage sites

100 ng of DNA was incubated with 2.5 U of each repair enzyme, APE1, Endo III, Endo IV, Endo VIII, hAAG, Fpg and UDG (and a mixture of these enzymes that constitute the ‘enzyme cocktail’) in 1× CutSmart buffer and incubated for 1 h at 37°C. The *in vitro* DNA repair was followed by incubation with dNTPs (1 μM of dATP, dGTP, dCTP, 0.25 μM dTTP and 0.25 μM aminoallyl-dUTP-ATTO-647N) in 1× NEBuffer 2 and DNA polymerase I (1.25 U) for 1 h at 20°C. The reaction was terminated with 2.5 μL of 0.25 M EDTA (Sigma–Aldrich).

#### Silanization of coverslips

Glass coverslips were functionalized as follows: 18 × 18 mm glass coverslips were placed in a coverslip rack which was carefully submerged in an acetone solution containing 1% APTES and 1% ATMS (39,40). The activated coverslips were rinsed with 2:1 (*v/v*) acetone:water solution and dried under a nitrogen gas flow right before DNA stretching and imaging experiments.

#### DNA staining and imaging

Fluorescently labelled DNA was diluted in 0.5× TBE and stained with 320 nM YOYO-1 in a total volume of 50 μL. To prevent photobleaching, 2% BME was added prior to image acquisition. The stained DNA sample (3.2 μL) was put at the interface of a silanized coverslip and a clean microscopy slide. The extended DNA molecules were imaged with a fluorescence microscope (Zeiss Observer.Z1) using an Andor iXON Ultra EMCCD camera equipped with a Colibri 7 LED illumination system. Band-pass excitation filters (475/40 and 640/30 nm) and bandpass emission filters (530/50 and 690/50 nm) were used for YOYO-1 and aminoallyl-dUTP-ATTO-647, respectively.

#### Data analysis and statistics

A customed-made MATLAB software was used to analyse the data. The total number of colocalized aminoallyl-dUTP-ATTO-647N labels (dots) was divided by total DNA length and expressed as Dots/MBp. The relative extension of DNA on the cover slips was estimated by stretching lambda DNA (48,502 bp, New England Biolabs) in a similar buffer to determine 1 μm stretched DNA to be ∼3000 bp. The software excludes fluorescent labels at the end of the DNA strand which could have resulted from DNA strand breaks while handling the samples after extraction from the PBMCs. Overlapping DNA strands were also excluded from the analysis. Experiments were performed in technical duplicates and analysed in Graphpad prism (10.1.1). One-way ANOVA (analysis of variance) statistical significance was determined using Tukey’s model for multiple comparisons with a family-wise alpha threshold and confidence level of 95% (confidence interval). *P*-values are represented using the GP style; *P ≤ 0.0332; **P ≤ 0.0021; ***P ≤ 0.0002; ****P < 0.0001.

### NCI-60 analysis

The copper(II)-free ligand, BPL-C6, was submitted to the National Cancer Institute (NCI) Developmental Therapeutics Program (DTP) where the cytotoxicity profile was investigated across the 60 human cancer cell line panel according to the sulforhodamine B protocol (Supplementary Section S-8 and Supplementary Figures S25 and S26) (41,42). The COMPARE algorithm was employed to evaluate the similarities (Pearson correlation) of BPL-C6 with standard agents using publicly available datasets (https://dtp.cancer.gov/databases_tools/compare.htm). Data was plotted in GraphPad Prism (10.1.1).

### Viability assessment

The MDA-MB-231, MDA-MB-468 cell lines were obtained from American Type Culture Collection. The BT-549 cell line was obtained from Cell Line Services (now Cytion). MDA-MB-231 and MDA-MB-468 cells were grown in Dulbecco’s modified Eagle medium (Sigma–Aldrich), supplemented with 10% fetal bovine serum (FBS) (ThermoFisher) and 1% penicillin–streptomycin (P/S) (ThermoFisher). BT-549 cells were grown in Roswell Park Memorial Institute (RPMI) 1640 (ThermoFisher) supplemented with 10% FBS and 1% P/S. All cells were cultured in flasks at 37°C in a humidified incubator with 5% CO_2_. For viability assessment, the protocol was adjusted according to the sulforhodamine B protocol from the NCI. The cells were seeded (5.0 × 10^3^ cells for MDA-MB-231 and MDA-MB-468 cells and 2.5 × 10^3^ cells for BT-549 cells) in 96-well plates. The following day, the cells were exposed to either vehicle (DMSO; amount equivalent to highest complex concentration), Cu(NO_3_)_2_ control (concentration equivalent to highest complex concentration) and free ligand (ligand) or complex (Cu_2_-BPL-C6) in the following concentrations: 1.62–6.29 μM for the MDA-MB-468 cell line and 7.86–30 μM for the remaining cell lines. After 48 hours of incubation, viability was assessed using the RealTime-Glo (Promega) according to manufacturer’s instructions (end-reading protocol) using a GloMax Explorer (Promega). Viability was normalized to vehicle-treated cells and presented as percentages. Data are presented as mean ± standard error of the means and Student’s two-tailed *t*-test was used to determine statistical significance. *P*-values were defined as follows: *P < 0.05, **P< 0.01 and ***P < 0.001.

## Results and discussion

### Cu_2_-BPL-C6 synthesis

**Figure 2.**
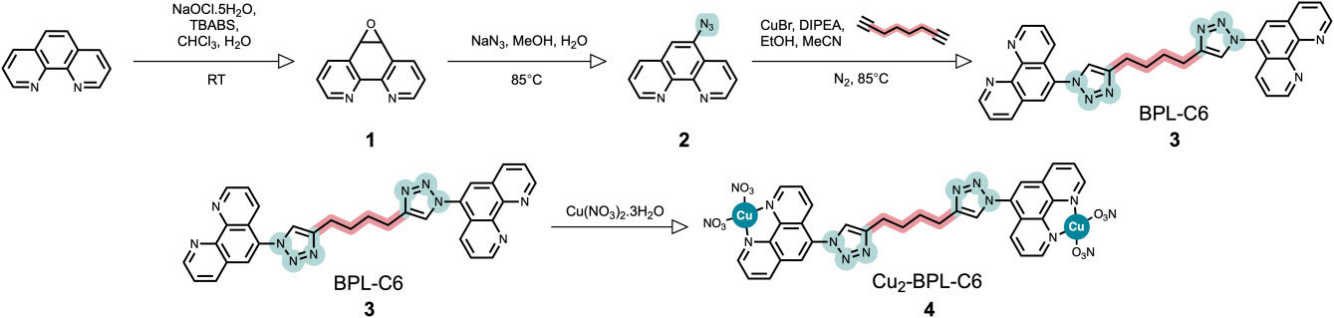
Synthetic route for the generation of Cu_2_-BPL-C6.

Initially, 1,10-phenanthroline was converted to an epoxide intermediate, **1**, followed by conversion to an azide, **2**. CuAAC click chemistry was then employed to synthesize BPL-C6, **3**. The azide-bearing phenanthroline ligand **2** was ‘clicked’ to a 1,7-octadiyne linker in a 2:1 (azide:alkyne) ratio with 1,7-octadiyne (Figure 2). Next, BPL-C6 was treated with two equivalents of copper(II) nitrate trihydrate to generate the active Cu(II)-metallodrug, **4**. The ligand, **3**, was synthesized while shielded from light at a high temperature (85°C) using an excess of a copper(I) species, CuBr, under inert atmosphere (N_2_), and supplemented with catalytic DIPEA. A slight excess of 2 (2.1 equivalents) was used to ensure the complete consumption of the alkyne. The crude material was treated with 0.5 M EDTA for 2 h to yield a beige precipitate which was extracted using CHCl_3_ and recrystallized from methanol, yielding a pure product (Figure 2). The ligand was successfully crystallized using slow vapour diffusion and verified using single-crystal XRD (Supplementary Section S-5 and Supplementary Figures S16–S20). In addition to the generation and characterization of the complex, an *in-situ* Cu(II) binding study was performed whereby ratios of BPL-C6:Cu(II) ranging from 1:1 to 1:10 were co-incubated. In all cases, the formation of a discrete cationic complex, [(C_32_H_24_)Cu_2_(NO_3_)_3_]^+^ at 860.04 *m/z* (Supplementary Section S-4 and Supplementary Figures S11–S13), was observed. Solutions containing BPL-C6 and two equivalents of Cu(II), are henceforth referred to as Cu_2_-BPL-C6.

### DNA-binding experiments

**Figure 3.**
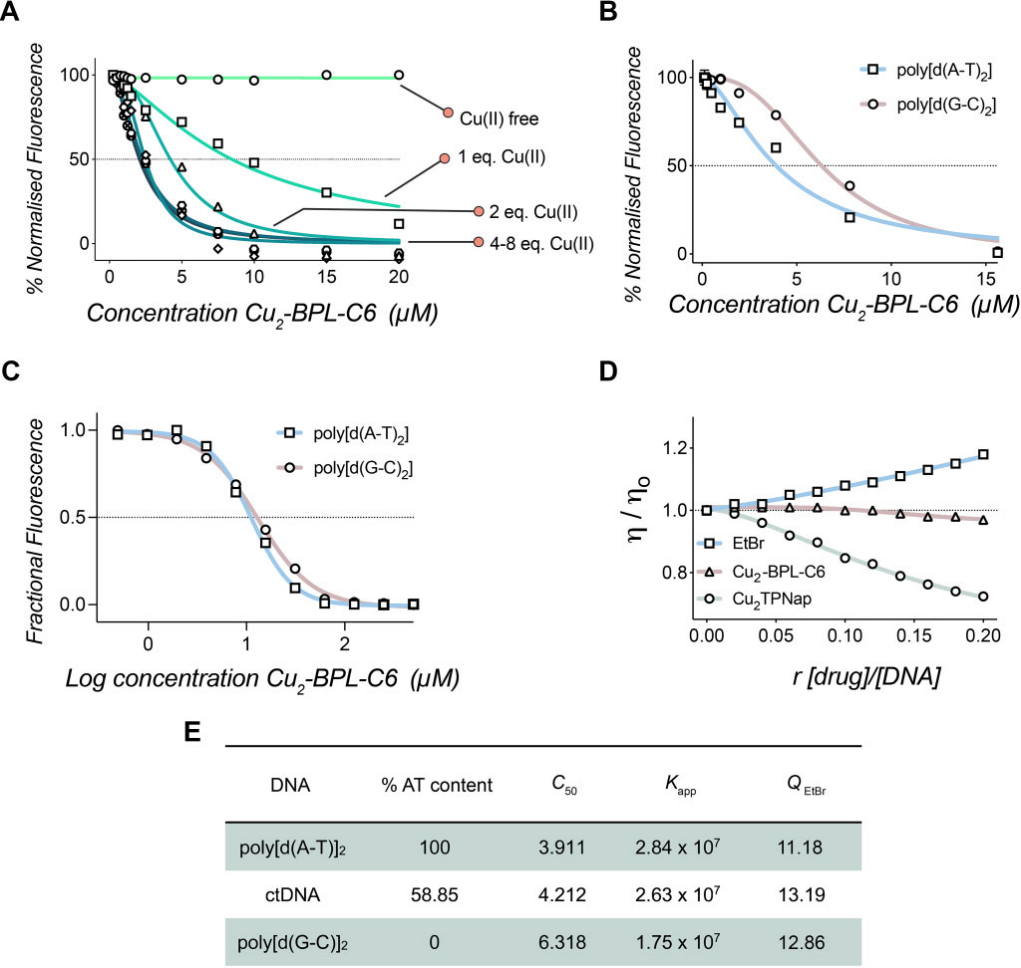
(**A**) EtBr displacement assay with BPL-C6 in the presence of titrated copper(II). (**B**) Binding profile of Cu_2_-BPL-C6 with poly[d(A-T)_2_] and poly[d(G-C)_2_], determined by the displacement of EtBr. (**C**) Quenching of limited bound EtBr bound to synthetic copolymers of poly[d(A-T)_2_] and poly[d(G-C)_2_] by Cu_2_-BPL-C6. (**D**) ctDNA viscosity profile of Cu_2_-BPL-C6 along with EtBr and Cu_2_TPNap controls (29,43). (**E**) C_50_ values for EtBr fluorescence quenching upon titration of Cu_2_-BPL-C6 2:1; Apparent binding constants of Cu_2_-BPL-C6 with poly[d(A-T)_2_], ctDNA and poly[d(G-C)_2_]; Q-value (EC_50_) of Cu_2_-BPL-C6 with ctDNA poly[d(A-T)_2_] and poly[d(G-C)_2_] (EtBr limited-bound).

The DNA binding efficacy of Cu_2_-BPL-C6 was monitored using an EtBr displacement assay (29). ctDNA was saturated with EtBr—a well-known intercalator—which enables the indirect, or apparent, DNA binding (*K*_app_) to be calculated upon the ejection of EtBr by a competitor (i.e. Cu_2_-BPL-C6). To determine the optimum ratio of BPL-C6 to copper, a Cu(II) concentration study was performed using this assay, with BPL-C6:Cu(II) ratios, ranging from 1:0 to 1:8 (Figure 3A). The scaffold in the absence of Cu(II) showed no appreciable depletion in EtBr fluorescence, indicating that BPL-C6 binds poorly to ctDNA. When Cu(II) is introduced with the ligand, a dose-dependent depletion in fluorescence was observed at 590 nm. Fluorescence depletion was monitored from ratios 1:0–1:6 [BPL-C6:Cu(II)]. Interestingly, the apparent binding constant of BPL-C6:Cu (II) 1:2, which corresponds to the maximum coordination number, yielded a high apparent binding constant (*K*_app_ = 2.63 × 10^7^ M^−1^) and compares favourably to several other reported dinuclear Cu(II) complexes (16,43). ctDNA binding studies were also performed in the presence of several other metals including Zn(II), Mn(II), Fe(II), Co(II) and Ni(II) and it was observed that Cu(II) showed the most promising DNA-binding affinity (Supplementary Section S-7 and Supplementary Figure S22).

Next, the binding preference of Cu_2_-BPL-C6 was investigated for adenine-thymine (AT)-rich and guanine-cytosine (GC)-rich DNA, along with ctDNA (summarized in Figure 3E). Synthetic co-polymers of poly[d(A-T)_2_] and poly[d(G-C)_2_] were elected as DNA substrates for binding experiments with Cu_2_-BPL-C6 (Figure 3B and C). Both sequences were examined using a 1:2 ratio of BPL-C6:Cu(II), and yielded apparent binding constant values for poly[d(A-T)_2_] (*K*_app_ = 2.84 × 10^7^ M^−1^) and poly[d(G-C)_2_] (*K*_app_ = 1.75 × 10^7^ M^−1^) (Figure 3B).

EtBr fluorescence quenching experiments were performed to determine if Cu_2_-BPL-C6 preferentially binds and quenches limited bound EtBr from AT-rich or GC-rich DNA sequences. The quenching of EtBr to these synthetic co-polymers permits the identification of preferential DNA binding sites (29). This preference is calculated by fitting the Hill model to the data, and measuring the EC_50_ of the interaction, which in this instance is known as the quenching value (*Q*) (29). Here, Cu_2_-BPL-C6 was introduced to poly[d(A-T)_2_] and poly[d(G-C)_2_] at concentrations ranging from 0.49 to 500 μM. Quenching values of 11.18, and 12.86 μM were recorded for poly[d(A-T)_2_] and poly[d(G-C)_2_], respectively (Figure 3C). This indicates that Cu_2_-BPL-C6 has little preference for quenching EtBr bound to AT- or GC-rich DNA and indicates the potential for a combination of major and minor groove binding modes, which is supported by the quenching data of ctDNA (Supplementary Section S-7 and Supplementary Figure S24).

DNA binding was then directly investigated using the relative change in viscosity (*η/η_0_*) of DNA upon drug-binding. Classical DNA intercalators, such as EtBr, increase DNA viscosity (*η*) (Figure 3D), whereas some dinuclear Cu(II) major groove binders, such as Cu_2_TPNap (43) (Figure 3D), show a decrease in viscosity due to DNA condensation effects. The relative change in viscosity upon addition of Cu_2_-BPL-C6 is not appreciable and differs from that of classical DNA intercalators and the Cu_2_TPNap complex. [Co (NH_3_)_6_]Cl_3_, which is known to electrostatically interact with DNA shows a negative viscosity trend, due to charge neutralization and compaction effects. In comparison, Cu-Phen_2_—a compound that semi-intercalates in the minor groove—results in a positive viscosity profile upon successive titration. Netropsin—a minor groove binder—does not appreciably change DNA viscosity (8,29) and is thus accommodated by B-DNA without impacting structural dynamics. Similarly, pentamidine—another minor groove binder—shows a very slight decrease in viscosity upon titration with ctDNA. This suggests Cu_2_-BPL-C6 has a different binding mode compared to the simple Cu-Phen_2_ monomer (30), and it is not possible (based on this data alone) to rule out either a minor groove binding mode, or competing heterogenous binding modes such as condensation combined with intercalative interactions, producing such a viscosity profile.

### CD experiments

**Figure 4.**
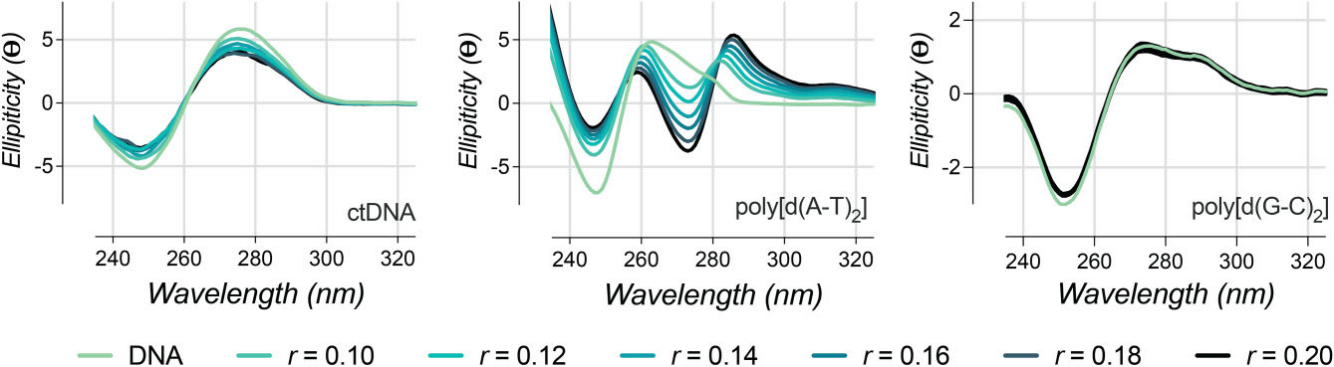
Cu_2_-BPL-C6 interaction with ctDNA (left), poly[d(A-T)_2_] (middle) and poly[d(G-C)_2_] (right) monitored using CD spectroscopy between *r* = 0 and *r* = 0.2, where *r* = [Drug]/[DNA].

CD allows the visualization of conformational changes of DNA upon recognition of a compound (16). B-DNA exhibits characteristic signals in ellipticity at 246 nm (negative) and 268 nm (positive) which correspond to helicity and base-pair stacking interactions, respectively. Monitoring the change in these characteristic signals upon the addition of Cu_2_-BPL-C6 informs how this complex introduces conformational changes in the DNA structure. Cu_2_-BPL-C6 and its interaction with ctDNA, poly[d(A-T)_2_], and poly[d(G-C)_2_] was investigated by CD spectroscopy at six concentration ratios of [drug]/[DNA] *r* = 0.1–0.2. Small increments of complex were selected for each experiment to avoid high levels of DNA condensation. With ctDNA, Cu_2_-BPL-C6 produces a minor increase at 246 nm, corresponding to changes in helicity, and a small decrease in the signal at 268 nm, corresponding to changes in base-pair stacking interactions (Figure 4). The change in CD for poly[d(A-T)_2_] is much more pronounced with a significant increase in ellipticity at 246 nm. A hypsochromic shift of the signal at 268 nm is also observed, corresponding to significant base stacking interactions. At 273 nm, a new CD signal emerges that displays increasingly negative ellipticity upon increasing concentrations of Cu_2_-BPL-C6. Additionally, another new CD signal at 286 nm, which becomes increasingly positive, is observed at a ratio of *r* = 0.1, and this signal shifts in a bathochromic manner up to *r* = 0.2. Cu_2_-BPL-C6-bound poly[d(A-T)_2_] also displays a slight increase at 314 nm compared to the untreated control, which—along with the signal at 286 nm—appears consistent with induced CD effects (44). This is indicative of an achiral molecule exhibiting a chiral signal once bound to DNA, due to the coupling of the complex with AT base pairs. Incubation of Cu_2_-BPL-C6 with poly[d(G-C)_2_] induces significantly less changes than that with poly[d(A-T)_2_]; there is a small increase in ellipticity at 246 nm and a small decrease in elliptical signal at 268 nm, which is similar to the profile for ctDNA. From these observations it is apparent that Cu_2_-BPL-C6 induces a higher degree of structural perturbation in AT-rich DNA.

### Fluorescence melting with FRET-labelled hairpin DNA

**Figure 5.**
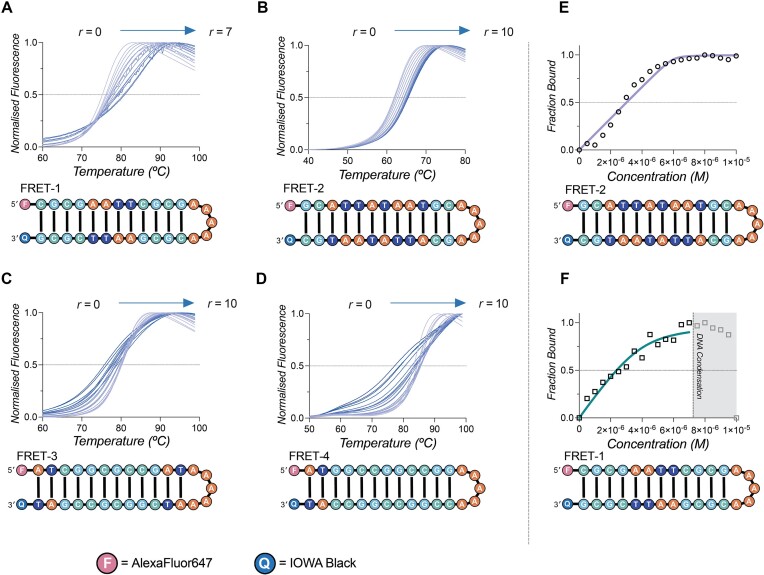
Cu_2_-BPL-C6 interaction with FRET labelled DNA hairpins (**A**) FRET-1 (**B**) FRET-2 (**C**) FRET-3 and (**D**) FRET-4 with increasing concentrations of complex. Bard analysis of FRET labelled DNA hairpins (**E**) FRET-2 and (**F**) FRET-1 at increasing concentrations of Cu_2_-BPL-C6.

To probe the DNA recognition properties of Cu_2_-BPL-C6 further, fluorescent melting experiments were performed with a series of DNA hairpins. These hairpins have varying compositions of AT and GC base-pairs and are labelled with 5′-AlexaFluor647 (F) and 3′-IOWA Black (Q) (Figure 5). All sequences were monitored at ratios of *r* = 0–10 where *r* = [drug]/[DNA], excluding FRET-1, where DNA condensation was observed *r* = >7 (Figure 5). Initially, Cu_2_-BPL-C6 was incubated with FRET-1 (a hairpin containing the Dickerson–Drew sequence), to determine the melting profile a mixed DNA sequence. The melting temperature of FRET-1 in the absence of complex was 74.9°C and a stepwise increase was observed upon sequential addition of Cu_2_-BPL-C6. Next, hairpin DNA containing a TATA sequence (FRET-2) and GC-rich sequences (FRET-3, FRET-4) were employed to evaluate the effect of DNA sequence interactions. FRET-2 had a T_M_ of 61.5°C, and similar to FRET-1, an increase in melting temperature was observed upon addition of Cu_2_-BPL-C6. GC-rich hairpins (FRET-3 and FRET-4) displayed an initial increase in thermal stabilization (+0.48°C at *r*= 2 and +1.21°C at *r* = 2.5, respectively) but subsequent destabilization and condensation at higher concentrations (Table 1). Overall, Cu_2_-BPL-C6 stabilizes DNA sequences containing AT base-pairs, while destabilization and condensation effects are observed for the GC-rich hairpin sequences.

**Table 1.** Fluorescent melting properties of FRET-labelled palindromic dodecamers FRET-1, FRET-2, FRET-3 and FRET-4 in the presence of Cu_2_-BPL-C6 at an *r* loading of 10 where *r* = [Cu_2_-BPL-C6]/[DNA]

DNA hairpin		FRET-1	FRET-2	FRET-3	FRET-4
DNA (control)	T_M_ (°C)	74.9	61.5	79.7	83.0
Cu_2_-BPL-C6	T_M_ (°C)	79.4^a^	65.1	76.0	76.7
	Δ T_M_ (°C)	+ 4.5^a^	+ 3.6	- 3.7	- 6.3

F = AlexaFluor647 Q = IOWA Black

^a^FRET-1 maximum *r* loading = 7

Next, the fraction bound hairpin was determined by normalizing the T_M_ at each respective *r* value and plotting these normalized T_M_ against Cu_2_-BPL-C6 concentration. The Bard equation was subsequently employed to fit the data (18,45). For FRET-1, a *K*_b_ of 1.06 × 10^7^ M^−1^ and an occupancy of two molecules per hairpin was determined, while a *K*_b_ of 4.38 × 10^8^ M^−1^ with an occupancy of three molecules per hairpin was obtained for FRET-2 (Figure 5E and F). For both FRET-3 and FRET-4 an actively decreasing bound fraction was found due to DNA condensation, and both profiles could not be fitted using the Bard model (Supplementary Section S-9 and Supplementary Figure S27). These observations corroborate the preference for binding with TATA DNA, which accommodates a larger number of Cu_2_-BPL-C6 molecules.

### MST experiments

**Figure 6.**
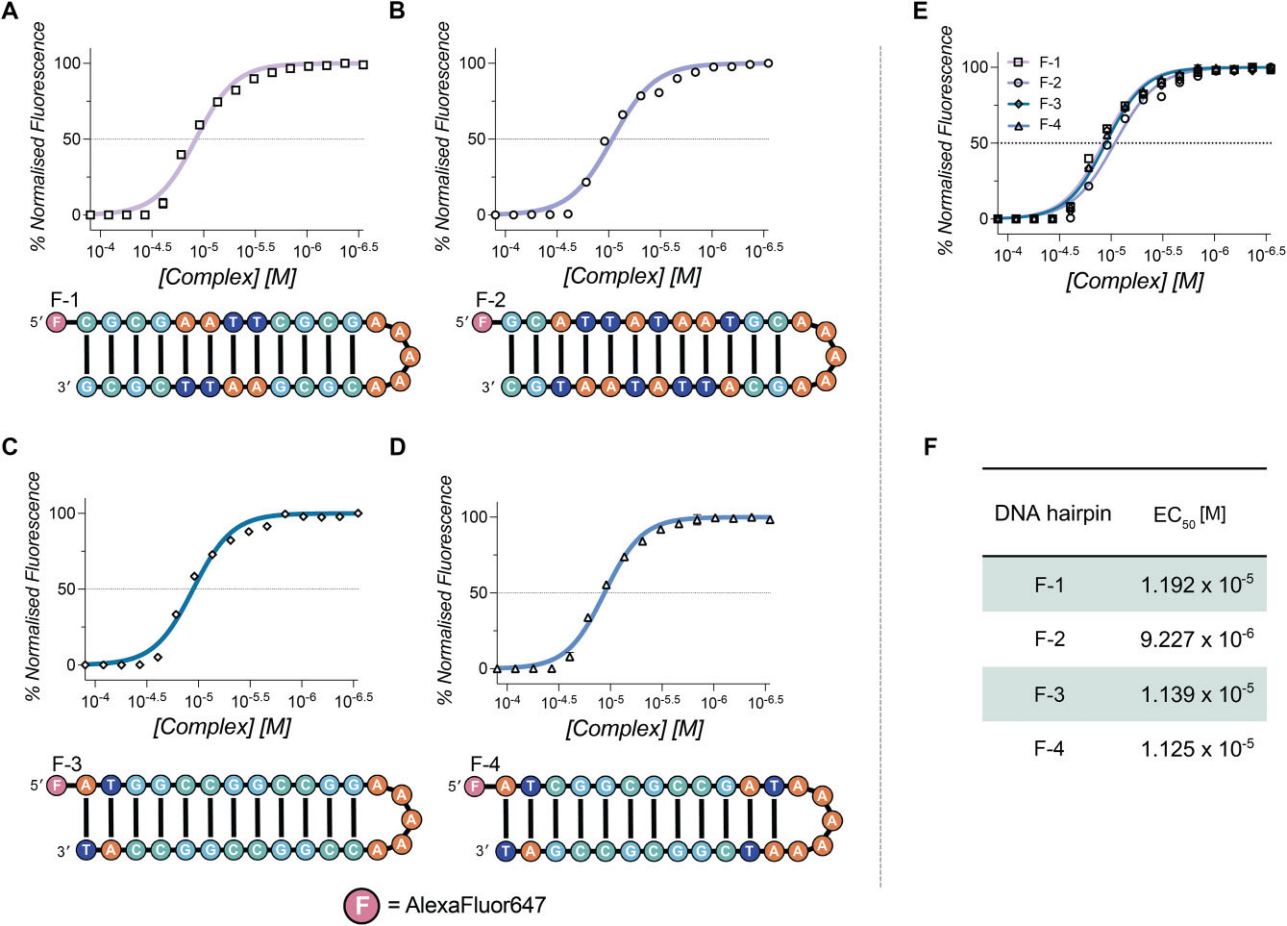
Normalized initial fluorescence data obtained from MST experiments with (**A**) F-1, (**B**) F-2 (**C**) F-3 and (**D**) F-4, all labelled with 5′-Alexafluor647. (**E**) Overlay of DNA hairpins analysed with MST. (**F**) Table indicating the EC_50_ values obtained [M] for the interaction of Cu_2_-BPL-C6 with each DNA hairpin.

MST is a technique that facilitates the direct measurement of a compound binding with target DNA. The interaction of Cu_2_-BPL-C6 with DNA was analysed using this technique, with DNA hairpins of the same sequence as those described above evaluated, but without the 3′-IOWA Black modification (18,31). A decrease in initial fluorescence of the labelled DNA was observed upon exposure to increasing concentrations of the complex. This dose-dependent decrease in fluorescence signal is indicative of DNA condensation, and has been observed with previous polynuclear Cu(II) scaffolds (32). DNA condensation prevented MST analysis and subsequently required the EC_50_ of the initial fluorescence signal to be obtained and analysed. Data were analysed using the Hill model (31) where F-1 (mixed AT and GC), F-3 (GC-rich) and F-4 (GC-rich) all revealed similar EC_50_ values in the order of low 10^–5^ M (Figure 6), while the EC_50_ value for F-2 (TATA sequence) was 9.2 × 10^–6^ M. This further supports the binding preference of Cu_2_-BPL-C6 to TATA regions of DNA.

### Cu_2_-BPL-C6 mediates DNA damage *via* superoxide and hydrogen peroxide oxidative pathways

**Figure 7.**
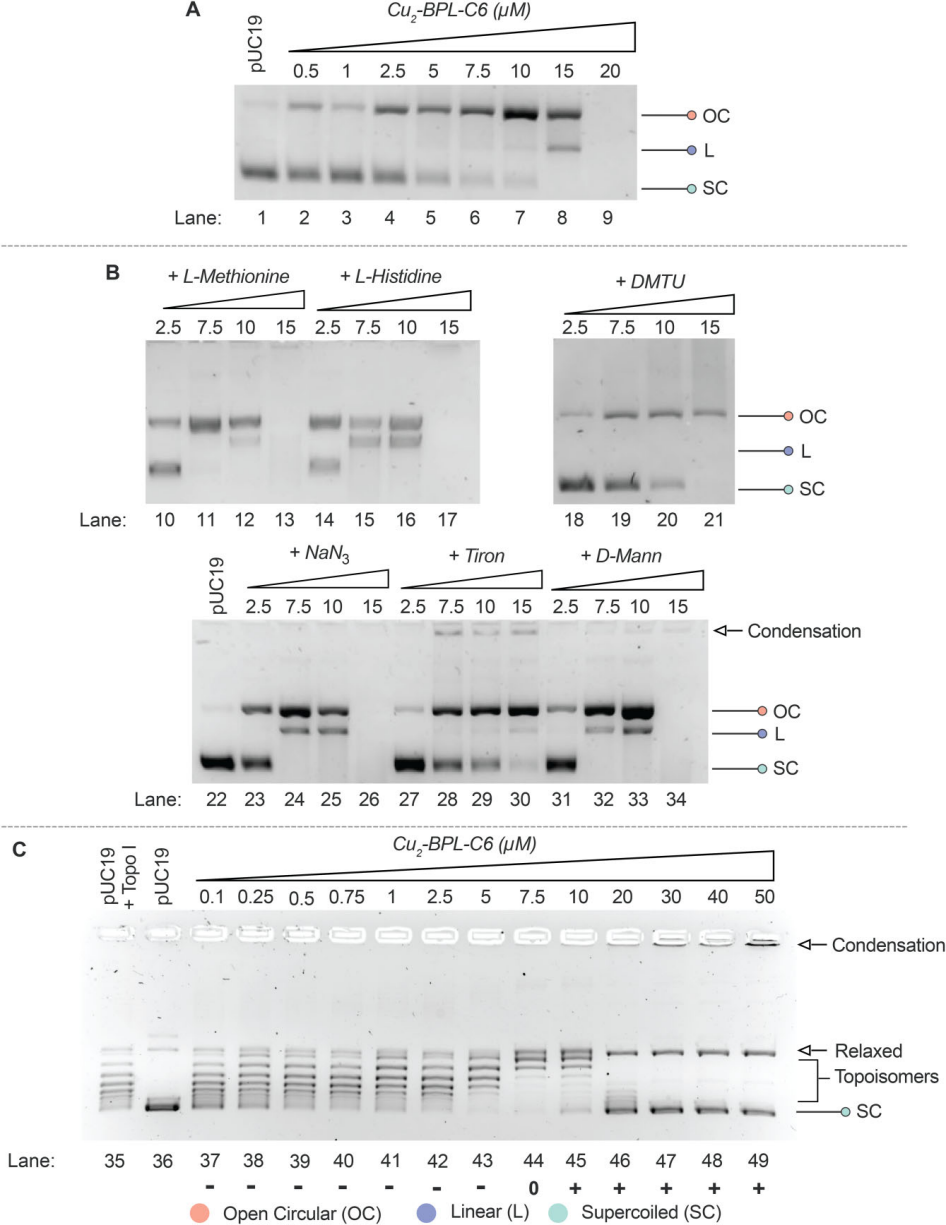
(**A**) Cleavage profile for supercoiled pUC19 DNA in the presence of Cu_2_-BPL-C6. (**B**) DNA cleavage profile of Cu_2_-BPL-C6 in the presence of scavengers DMTU, NaN_3_, tiron and *D*-mannitol, *L*-methionine and *L*-histidine. At the highest tested complex concentration in the presence of tiron, all three forms of pUC19 are present (lane 30) and DNA condensation is observed. In comparison, the control lane at the same concentration (lane 8) has no observable SC DNA. (**C**) Topoisomerase I inhibition by Cu_2_-BPL-C6, displaying negatively supercoiled DNA (lanes 37–43) and positively supercoiled DNA (lanes 45–49) together with evidence of DNA nicking.

Next, the metallo-nuclease activity of Cu_2_-BPL-C6 was evaluated. Plasmid DNA cleavage studies were performed in the presence of reductant (Na-*L*-ascorbate) and show efficient AMN activity (Figure 7A, lanes 1–9). Open circular (OC) DNA emerges upon exposure to sub-micromolar concentrations (0.5 μM) of the complex (Figure 7A, lane 2) and the linear (L) form appears in the presence of 10 μM of Cu_2_-BPL-C6 (Figure 7A, lane 7). Cu_2_-BPL-C6 is therefore more potent than previously studied Cu(II) salts (46), which require higher concentrations and only partially convert supercoiled (SC) DNA to the OC form.

Next, a radical scavenger assay was employed to determine the species responsible for DNA cleavage in the presence of exogenous reductant (ascorbate) (17,18,46,47). A range of radical scavengers were selected comprising *L*-methionine (HOCl), *L*-histidine (^1^O_2_), DMTU (H_2_O_2_), NaN_3_ (^1^O_2_), tiron (O_2_^•-^) and *D*-mannitol (^•^OH) (Figure 7B, lanes 10–34). DNA cleavage was inhibited in the presence of tiron and DMTU compared to the control, indicating that the primary species generated during the DNA cleavage process are superoxide—sequestered by tiron—and hydrogen peroxide—sequestered by DMTU—suggesting that superoxide or a metallo-superoxo intermediate is chiefly responsible for plasmid DNA cleavage (Figure 7C, lanes 18–21, 27–30). Interestingly, NaN_3_, *D*-mannitol, *L*-methionine and *L*-histidine all enhance the DNA cleavage profile of Cu_2_-BPL-C6. This enhancement in activity may stem from the inhibition of other redox reactions that do not yield oxidative DNA damage products, thereby promoting the active superoxide pathway. The superoxide-mediated DNA damage pathway has been previously associated with a number of copper AMNs (16–18,46,48), and it has been found to be a mediator of *in cellulo* DNA damage with several cytotoxic dinuclear copper compounds (16).

We then employed a topoisomerase I (Topo I) unwinding assay to evaluate the intercalative properties of Cu_2_-BPL-C6 (49). Topo I is an enzyme that mediates relaxation of negatively supercoiled DNA into topoisomers, which are distinguishable as discrete bands using agarose electrophoresis. The presence of low concentrations of an effective intercalator (e.g. EtBr) relaxes the supercoiled plasmid DNA, and thus inhibits Topo I. Increasing the concentration of the intercalator then results in overwinding of the plasmid to form positively supercoiled DNA, which is not recognized by the Topo I enzyme (16). Low concentrations of Cu_2_-BPL-C6 (0.1–5 μM) had little effect on the activity of Topo I (Figure 7C, lanes 37–43) in comparison to the pUC19 topoisomerase I control lane (Figure 7C, lane 35). Upon addition of 7.5 μM Cu_2_-BPL-C6, the plasmid DNA is converted to its relaxed form (Figure 7C, lane 44) with subsequent addition of the complex >10 μM (Figure 7C, lanes 45–49) yielding positively supercoiled DNA associated with intercalative overwinding, along with the emergence of OC DNA due to single strand break formation (Figure 7C, lanes 46–49). This activity correlates with self-activation properties whereby oxidative DNA damage is promoted in the absence of exogenous reductant. Overall, these results indicate that Cu_2_-BPL-C6 intercalates pUC19 DNA at a low exposure of compound (7.5 μM), inhibiting the activity of Topo I. At higher loading of the complex, overwinding and nicking effects become evident.

#### Self-activated DNA damage experiments

**Figure 8.**
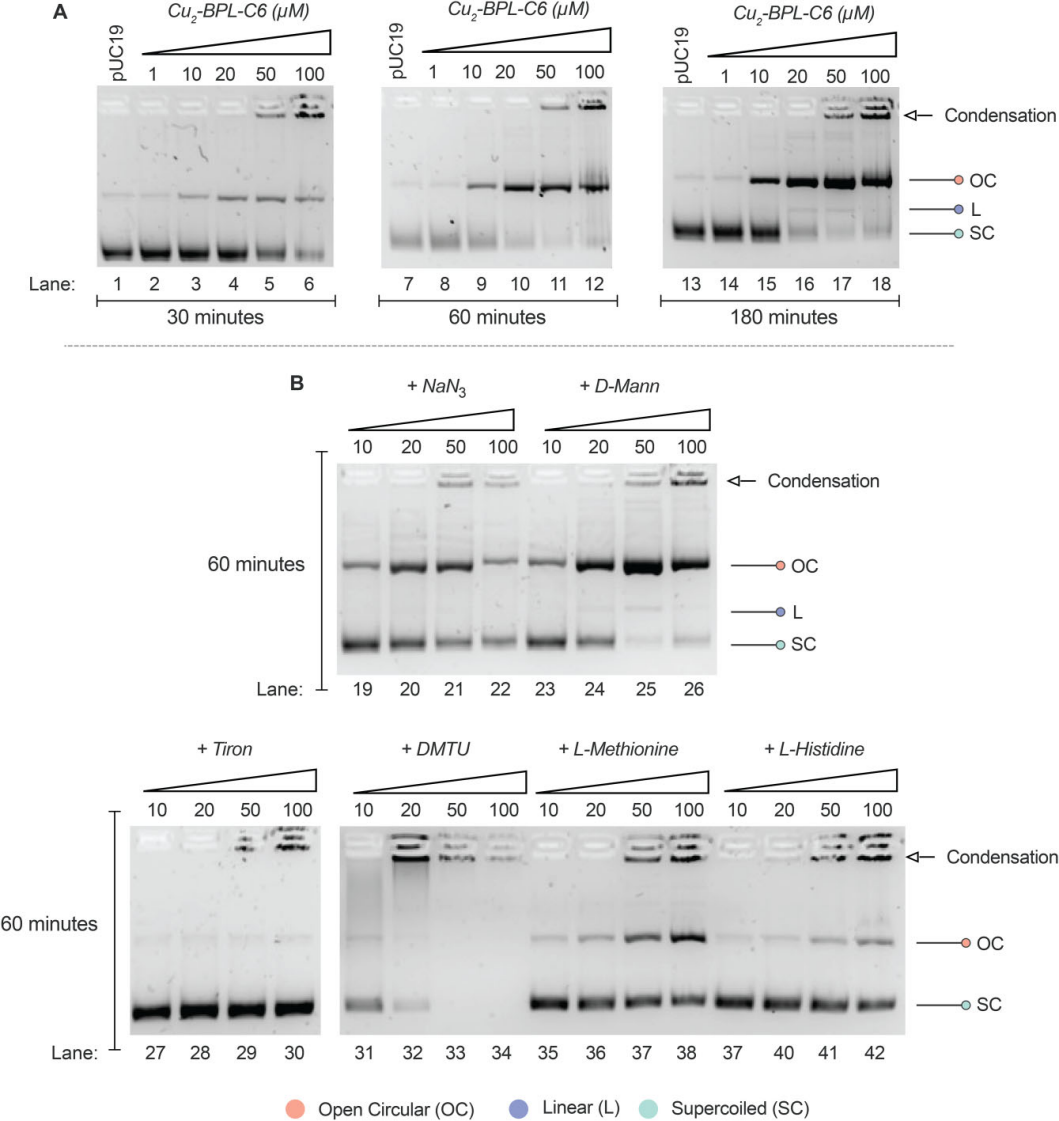
(**A**) Composite image of the self-activation profile of Cu_2_-BPL-C6 with supercoiled pUC19 DNA in the absence of reductant at 30, 60 and 180 min time points. (**B**) Self-activation ROS scavenger profile of Cu_2_-BPL-C6 in the absence of reductant with NaN_3_ and *D*-Mannitol, tiron, DMTU, *L*-methionine and *L*-histidine after a 60 min incubation.

Self-activating AMNs are characterized by their ability to promote oxidative DNA damage in the absence of co-activating reductants (20,21). A preliminary investigation of BPL-C6 with first-row metal ions, including Zn(II), Mn(II) and Cu(II), was performed. Results show that only Cu(II) yielded efficient DNA cleavage (Supplementary Section S-10 and Supplementary Figure S28). Therefore, the self-activation profile of BPL-C6 with Cu(II) was further investigated. The self-activating cleavage profile was monitored after 30, 60 and 180 min at 37°C with supercoiled pUC19 DNA (Figure 8A). The complex begins to nick pUC19 at 10 μM (Figure 8A, lane 3) in the absence of ascorbate, and this conversion continues upon longer incubation. It was further observed that Cu_2_-BPL-C6 induces DNA condensation at concentrations >20 μM (Figure 8A, lanes 5–6, 11–12, 17–18).

The self-activated DNA damage mechanism was then investigated using the radical scavengers employed in earlier analysis. Compared to the self-activated cleavage control (Figure 8A, lanes 7–12), NaN_3_ appears to have little effect on the profile of the complex (Figure 8B, lanes 19–22). Interestingly, *D*-mannitol again appears to increase the cleavage activity and generates linear DNA upon exposure to 20 μM of the complex under these conditions (Figure 8B, lanes 24–25). Tiron completely inhibits DNA cleavage in the absence of reductant, suggesting superoxide is again responsible for mediating DNA damage (Figure 8B, lanes 27–30). In the presence of DMTU, DNA condensation is promoted with almost complete DNA condensation observed at a concentration of 20 μM (Figure 8B, lane 32), this enhanced activity differs from the inhibition of cleavage activity observed when the complex damages DNA in the presence of ascorbate, suggesting a potentially different mechanistic pathway. *L*-Methionine had little effect on the DNA cleavage profile compared to the control (Figure 8B, lanes 35–38) while *L*-histidine was observed to hinder activity (Figure 8B, lanes 37–42). Additional controls show that Cu-(Phen) and simple Cu(NO_3_)_2_ salts do not damage DNA under the same concentration range (Supplementary Section S-10 and Supplementary Figure S29).

### AFM for assessing DNA damage and condensation induced by Cu_2_-BPL-C6

To better understand and visualize the damage mechanisms and condensation effects on DNA during treatment with Cu_2_-BPL-C6, we performed in-liquid AFM experiments. In the presence of 1 mM Na-*L*-ascorbate we observe both a relaxation and linearization of the DNA molecules exposed to Cu_2_-BPL-C6 for 30 min (Supplementary Section S-11 and Supplementary Figure S30A). These effects are quantified to determine the proportion of circular or linear molecules. At 10 μM Cu_2_-BPL-C6, we observe 12% of the molecules in a linear form which rises to 100% at 15 μM Cu_2_-BPL-C6 treatment (Supplementary Section S-11 and Supplementary Figure S30B), indicating DSBs are present in all the DNA molecules. We also calculated the smallest bounding area of the circular DNA molecules, which provides a measure of relaxation. Comparing the untreated pUC19 to 10 μM Cu_2_-BPL-C6 treatment we also observe an increase in the smallest bounding area of the molecules (Supplementary Section S-11 and Supplementary Figure S30C), which is indicative of the presence of single-stranded breaks or other DNA damage that enable the DNA to adopt an open conformation.

**Figure 9.**
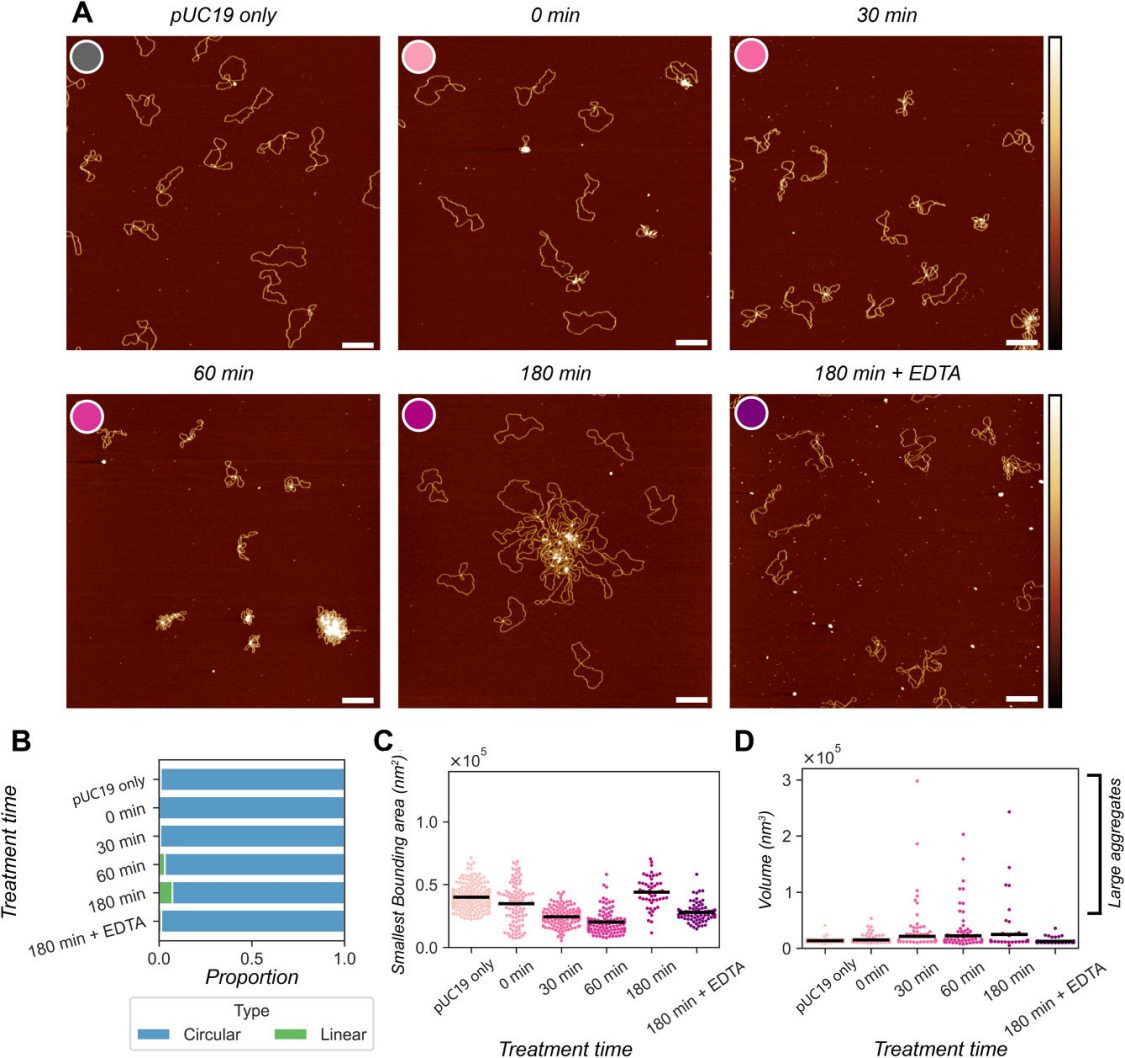
In-liquid AFM showing the self-activation activity of 20 μM Cu_2_-BPL-C6 on pUC19. (**A**) Representative AFM images of untreated pUC19, Cu_2_-BPL-C6 treated pUC19 after 0, 30, 60 and 180 min and the 180 min time point after the addition of 200 μM EDTA. Scale bars = 200 nm, height scales = −3 to 4 nm. (**B**) Quantification of the proportion of circular and linear molecules present in the images. (**C**) Quantitative analysis of the smallest bounding area of individual circular molecules. (**D**) The total volume of the masked grains. N-values are as follows: pUC19 only: 213; 0 min: 110, 30 min: 140, 60 min: 127, 180 min: 69, 180 min + EDTA: 79.

In the absence of reducing agent, we observe the self-activation effects of 20 μM Cu_2_-BPL-C6 on pUC19 plasmids with AFM between 0 and 180 min (Figure 9A). Quantification of the proportion of circular molecules (Figure 9B), smallest bounding area (Figure 9C) and grain volume (Figure 9D) was performed to quantify the formation of DSBs, other forms of DNA damage and condensation behaviour respectively. Initially, we observe little change in the DNA conformation compared to the untreated plasmid. However, at 30 and 60 min, intramolecular compaction of individual molecules was observed, and quantified by a decrease in bounding area (40% reduction between untreated and 60 min). At the same time points an increase in the number of large volume grains was observed, which indicates DNA condensation. After 180 min of treatment, there is an increase in the bounding area of individual molecules and number of linear molecules, indicating an increase in damaged DNA. There is also an increase in grain volumes indicating DNA condensation is still occurring. Finally, addition of 200 μM EDTA at the 180 min time point appeared to reverse the DNA condensation behaviour, with no high-volume grains observed.

For comparison, the same self-activation experiment was performed using 20 μM of Cu-Prodigiosin (Supplementary Section S-11 and Supplementary Figure S31A). Prodigiosin is a polypyrrole marine alkaloid with antimicrobial and anticancer properties (50,51). In the presence of copper(II), the complex is known to induce potent self-activating DNA damage (20). The proportion of linear and circular DNA was quantified (Supplementary Section S-11 and Supplementary Figure S31B) and a reduced intramolecular compaction effect was observed after 0 and 30 min of treatment, quantified by a 20% reduction in the bounding area between the untreated and 30 min treated samples (Supplementary Section S-11 and Supplementary Figure S31C). At 60 min of treatment onwards, the bounding area increased, as the DNA started to adopt more OC conformations. Low levels of DNA condensation were observed from 30 min onward (Supplementary Section S-11 and Supplementary Figure S31D), although the number of large aggregates was five times less than for the Cu_2_-BPL-C6 treatment. The effects observed for Prodigiosin and Cu_2_-BPL-C6 followed the same global trend on a comparative timescale, showing similar propensities for the formation of DSBs. The major difference was in the levels of intramolecular and intermolecular compaction which was much reduced for Prodigiosin compared to Cu_2_-BPL-C6.

### Apurinic and apyrimidinic sites form the majority of DNA damage lesions generated by Cu_2_-BPL-C6 in peripheral blood mononuclear cells

**Figure 10.**
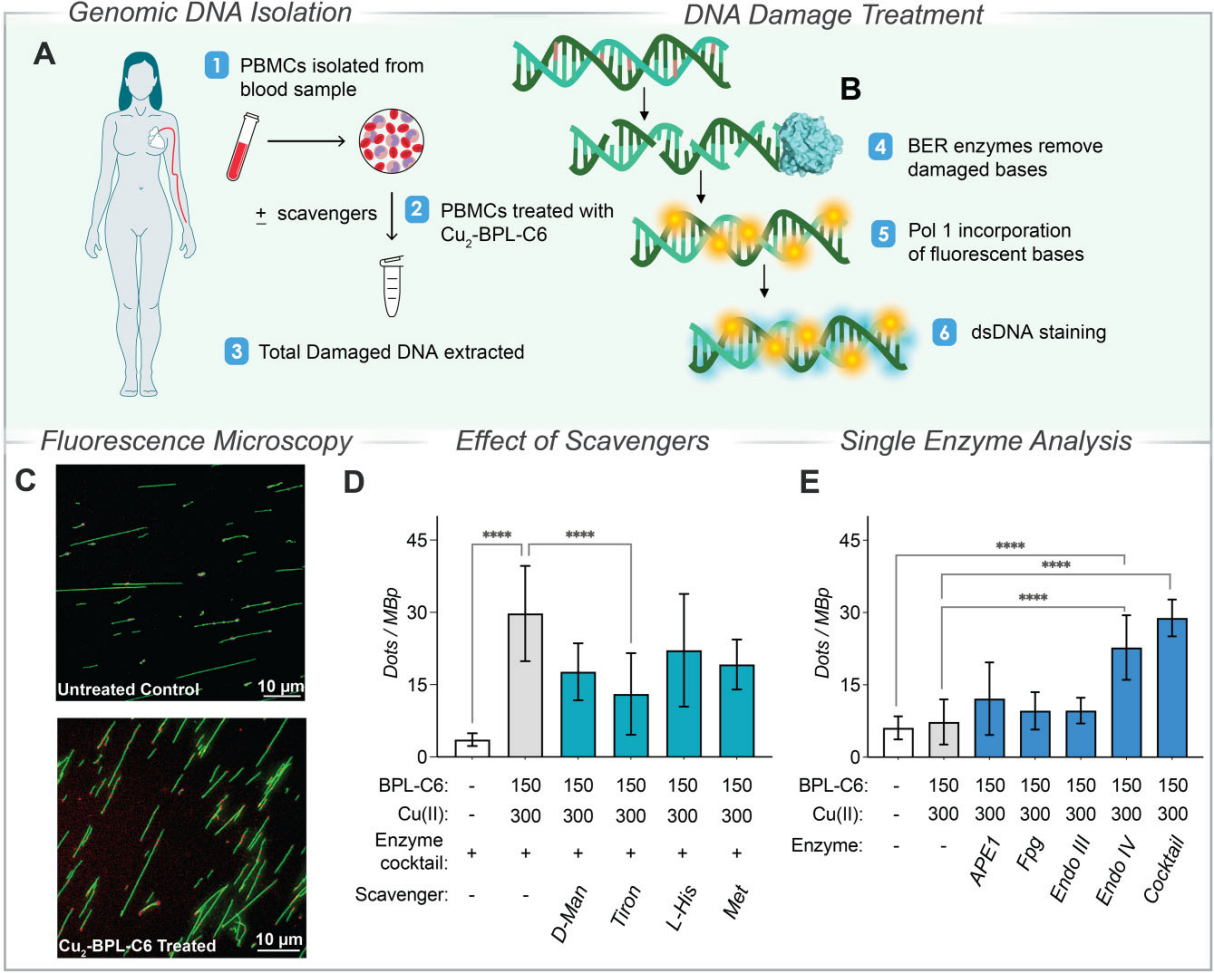
DNA damaging effect of Cu_2_-BPL-C6 on PBMCs. (**A**) Sample collection and treatment with Cu_2_-BPL-C6. (**B**) DNA repair with fluorescently labelled bases and post staining with YOYO-1 dye. (**C**) Microscopic images of control (untreated) DNA (top) and Cu_2_-BPL-C6 (150 μM) treated DNA (bottom) isolated from PBMCs (scale bar = 10 μm). (**D**) DNA damage detection in the presence of a repair enzyme cocktail for PBMCs treated with Cu_2_-BPL-C6 with and without antioxidant scavengers. (**E**) Identification of lesions generated by Cu_2_-BPL-C6 treated with and without BER enzymes, along with a combination thereof (cocktail).

To determine cellular damage caused by Cu_2_-BPL-C6, we used human peripheral blood mononuclear cells (PBMCs). PBMCs were selected as they are primary non-proliferating cells, making them an appropriate model for accurately representing DNA repair mechanisms. We performed experiments using a technique that employs nick translation coupled with single molecule imaging to detect single strand lesions (52). The assay involves the use of DNA repair enzymes which identify and excise the DNA lesion, preparing the DNA lesions for the action of DNA polymerase I, which incorporates fluorescently labelled nucleotides at the sites of damage. This assay has been used to detect DNA damage induced by ionizing radiation (38) and several chemotherapy drugs, including bleomycin (53) and etoposide (54). In this study, isolated PBMCs were treated with Cu_2_-BPL-C6 (150 μM) and the extracted DNA was incubated with base excision repair (BER) enzymes, which recognise base lesions and excise them. In a subsequent reaction step, dNTPs (aminoallyl-dUTP-ATTO-647N) were introduced with DNA polymerase I which leads to fluorescent base incorporation at the damage sites (Figure 10A and B). The DNA backbone was stained with YOYO-1 which was followed by stretching of the DNA molecules on silanized glass coverslips to quantify the level of single-stranded DNA damage at the single DNA molecule level. Damage is seen as fluorescent spots along the length of each DNA molecule (Figure 10C). When comparing the untreated control to Cu_2_-BPL-C6-treated cells in the presence of a cocktail of DNA repair enzymes, there was an ∼6-fold increase in observed signal due to DNA damage and subsequent repair (Figure 10D).

Next, PBMCs were incubated with radical scavengers *D*-mannitol (^•^OH), tiron (O_2_^•−^), *L*-histidine (^1^O_2_), *L*-methionine (HOCl) prior to the introduction of Cu_2_-BPL-C6 and this resulted in an overall decrease in DNA damage (Figure 10D). The highest inhibitory effect in DNA damage was observed in the presence of tiron, indicating that the *in cellulo* mechanism of DNA cleavage is likely mediated by superoxide, in agreement with the *in vitro* experiments. Next, to determine the type of DNA damage lesions formed, specific BER enzymes (APE1, Fpg, Endo III and Endo IV) were tested for their ability to recognize and remove damaged bases induced by Cu_2_-BPL-C6. The enzymes used recognize and act on different damage lesions (52,55). Endo III has a higher affinity for oxidized pyrimidines (56,57) while Fpg has higher affinity for oxidized purines, such as 8-oxoguanine (58). Endo IV shows increased activity for apurinic/apyrimidinic (AP) sites by directly cleaving AP sites (59) and has been previously shown to recognise and repair damage induced by bleomycin (53). The optimal concentration of each enzyme was experimentally derived to achieve maximal activity (53,54) allowing comparison between different enzymes. Here, Cu_2_-BPL-C6-induced damage relative to the untreated control was increased ∼4-fold for Endo IV, ∼2-fold for APE1, ∼1.5-fold for Endo III, and ∼1.5-fold for Fpg. Our data indicates that the cellular damage induced by Cu_2_-BPL-C6 is predominantly AP sites recognised by endonuclease Endo IV (Figure 10D). However, the data only indicates relative—rather than absolute—quantitative differences in the different types of damage, and we cannot exclude the presence of lesions that are not a substrate for the enzymes used. Finally, a further experiment to monitor the DNA damage of Cu_2_-BPL-C6 in the absence of BER enzymes was employed, yielding a similar signal to the control containing DNA only.

### *In vitro* anticancer screening with BPL-C6 and Cu_2_-BPL-C6

**Figure 11.**
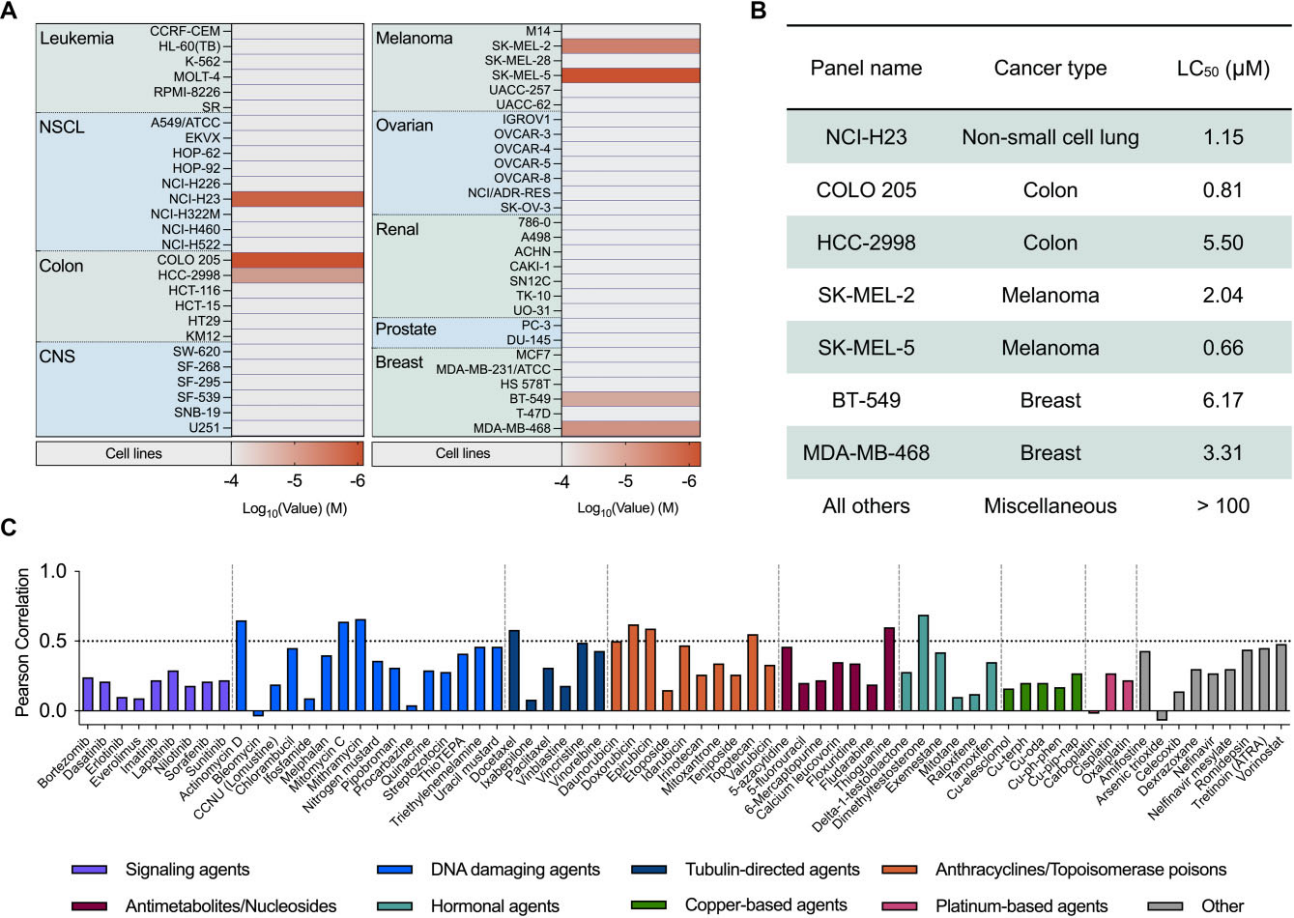
(**A**) NCI-60 panel results indicating the lethality of BPL-C6 with non-small cell lung cancers, colon cancers, melanoma and breast cancers. (**B**) Table of cell lines BPL-C6 shows activity toward. (**C**) COMPARE analysis of BPL-C6 with standard agents available on the NCI-60 COMPARE database, indicating their Pearson correlation using LC_50_ data. (Signalling agents, DNA damaging agents, tubulin-directed agents, anthracyclines/topoisomerase poisons, antimetabolites/nucleosides, hormonal agents, Cu(II) agents and others.)

To probe the *in cellulo* biological activity of the BPL-C6 ligand, we submitted the ligand to the NCI DTP for analysis. The cellular toxicity of BPL-C6 was initially screened at a preliminary single-dose point concentration, which was further progressed to a five-dose point concentration against a large panel of human cancer cell lines categorized according to their cancer-type (Figure 11A), listing the activity of BPL-C6 using 50% lethal concentration (LC_50_), total growth inhibition and 50% growth inhibition measurements (Supplementary Section S-8 and Supplementary Figures S25 and S26). The LC_50_ of BPL-C6 was measured with each cell line and is reported in the heat map generated in Figure 11A. The screening data indicates a high level of selectivity towards certain non-small cell lung cancers (NCI-H23), colon cancers (COLO 205, HCC-2998), melanoma (SK-MEL-2, SK-MEL-5) and breast cancers (BT-549, MDA-MB-468) (Figure 11B).

Using the NCI-60 COMPARE database and correlation function (https://dtp.cancer.gov/databases_tools/compare.htm), the LC_50_ activity of Cu(II)-free BPL-C6 was compared to a number of clinically relevant agents (Figure 11C). The results of these calculations present a swathe of data that can be used to draw some conclusions about the similarity of activity of BPL-C6 in comparison to a large dataset of existing compounds. BPL-C6 has similarities and a high (>0.5) Pearson correlation with DNA damaging agents actinomycin D, mitomycin C and mithramycin, docetaxel—a tubulin directed agent—anthracycline topoisomerase inhibitors doxorubicin and epirubicin, thioguanine—an antimetabolite—and the hormonal agent, dimethyltestosterone. Interestingly, there is a low Pearson correlation with bleomycin (60) and existing Cu(II)-based developmental chemotherapeutics available within the NCI-60 database.

**Figure 12.**
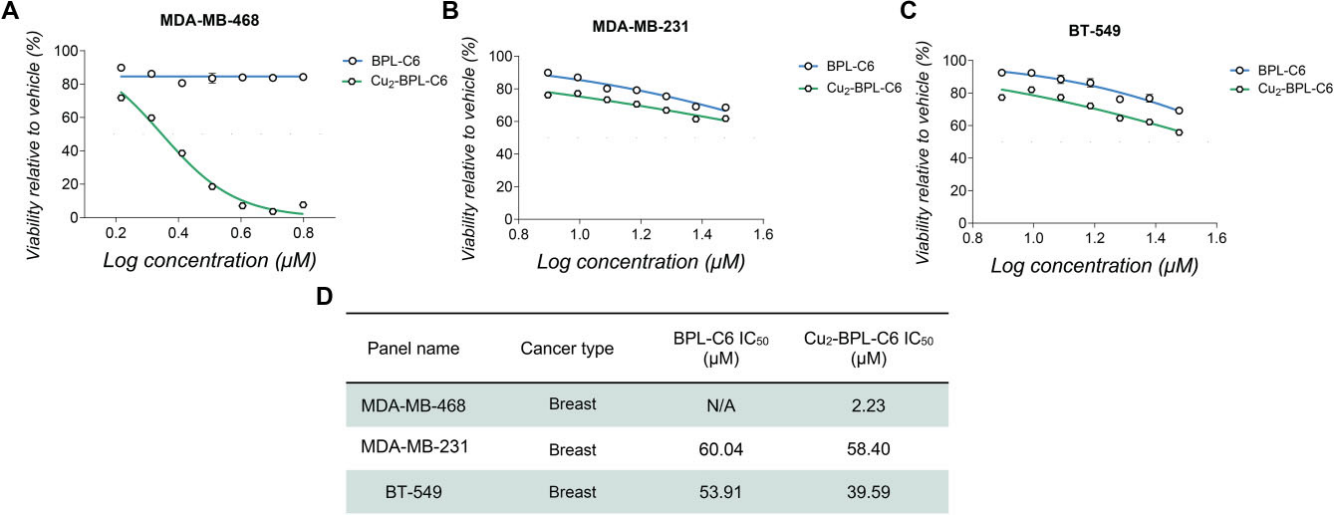
(**A**) Cellular viability assessment of BPL-C6 and Cu_2_-BPL-C6 with MDA-MB-468; (**B**) MDA-MB-231; (**C**) BT-549; (**D**) Table indicating IC_50_ data of BPL-C6 and Cu_2_-BPL-C6 with select cell lines.

To investigate specific differences in anticancer activity between the copper(II)-free ligand and the metal complex, further assessment was conducted with both BPL-C6 and Cu_2_-BPL-C6 in a head-to-head study with three human cancer cell lines: MDA-MB-468 (breast); MDA-MB-231 (breast) and BT-549 (breast) (Figure 12A–C). The most striking difference in activity between the copper(II)-free ligand and the metal complex was observed in the MDA-MB-468 cell line, where there is negligible activity with the free ligand, but potent effect on viability with the complex where it shows an IC_50_ value of 2.23 μM (Figure 12D). This result demonstrates that Cu(II) sensitises activity toward MDA-MB-468. The analysis also reveals that BT-549 is more active in the presence of Cu(II) while for MDA-MB-231, little difference in activity between the ligand and complex was found using this assay.

## Conclusion

The clinical relevance of anticancer metallodrugs has been constrained to activated bleomycin (60), platinum (61,62), ruthenium (63), gold (64) and, more recently, arsenic trioxide (Trisenox) (65–67). Copper compounds, such as the family of Casiopeínas^®^ (68), present an alternative approach to classical treatments by forming DNA damage lesions that are difficult to repair by endogenous enzymes, thus overcoming limitations associated with resistance (6). Click chemistry has not been widely employed to generate DNA damaging copper complexes (17,18), and this work established the preparation and biological properties of a new dinuclear copper complex called Cu_2_-BPL-C6. The BPL-C6 ligand was designed to force two redox-active copper centres—central for enhancing metallo-nuclease activity—to bind DNA *via* two 1,10-phenanthroline groups separated by a flexible aliphatic click chemistry linker. In this regard the complex differs from both Clip-Phen, where two 1,10-phenanthroline ligands are covalently bound to maintain a 2:1 ligand-to-metal ratio, and Cu-Oda, where two copper *bis*-1,10-phenanthroline centres are connected by a dicarboxylate linker.

Biophysical analysis of Cu_2_-BPL-C6 determined the apparent binding constant (*K*_app_) to ctDNA is significant (∼10^7^ M^−1^) and enhanced compared to other dinuclear Cu(II) complexes, such as Cu-Oda (∼10^5^ M^−1^) (69). The binding of BPL-C6 to DNA relies on Cu(II) ions, with preferential interactions at AT-rich tracts. To corroborate these findings, CD experiments showed large-scale conformational changes to poly[d(A-T)_2_] along with modest changes to ctDNA and poly[d(G-C)_2_]. Enhanced binding toward AT-rich DNA is borne out by fluorescence melting experiments with well-defined FRET-labelled hairpins. Here, a Dickerson–Drew based hairpin (FRET-1) and a TATA hairpin (FRET-2) recorded *K*_b_ values of 10^7^ M^−1^ and 10^8^ M^−1^, respectively. Conversely, experiments with GC-rich hairpins (FRET-3 and FRET-4) showed evidence of destabilization, consistent with condensation. Comparisons with recently reported minor groove binding tri-nuclear copper (II) complexes, TC-1 and TC-Thio (17,18), indicate that Cu_2_-BPL-C6 exhibits properties consistent with high-affinity recognition combined with sequence-selectivity for the TATA sequence. Overall, Cu_2_-BPL-C6 exhibits a heterogeneous DNA binding mode, combining groove binding, semi-intercalation and condensation effects attributed to its overall 4+ cationic charge. Semi-intercalation is evident in Topo I-mediated relaxation experiments, where the binding profile reveals both relaxation and overwinding of pUC19, aligning with the behaviour of classical intercalating agents. DNA cleavage experiments with the same plasmid (*vide infra*) highlight DNA damage induction (in the form of nicking) and condensation. We hypothesize that this unique interplay of interactions results in a distinctive viscosity profile, driven by competing intercalative and condensation forces (8,29).

In the presence of exogenous reductant, Cu_2_-BPL-C6 mediates extensive oxidative DNA damage in a mechanism consistent with state-of-art cleavage agents (16,70,18). The primary mode of damage is tightly linked to the generation of superoxide (or metal-superoxo species) and this mechanism is conserved in self-activated DNA cleavage reactions in the absence of reductant. Interestingly, the generation of single-strand breaks and DSB s by Cu_2_-BPL-C6 in absence of ascorbate occurs after a relatively short incubation time (180 min), which has only been seen in limited cases for other copper(II) complexes (21) and points to a facile redox mechanism. Deeper analysis of the oxidative mechanism showed that singlet oxygen is a key ROS species within self-activated DNA damage, but which is not involved in the cleavage mechanism with ascorbate. In this regard, Cu_2_-BPL-C6 shares similarities with Cu-Oda which exerts *in vitro* and *in cellulo* oxidative damage using a combination of superoxo- and singlet oxygen-mediated ROS. However, notable differences exist in their self-activated DNA damaging behaviour: Cu_2_-BPL-C6 damages pUC19 DNA within a shorter timeframe of 30–180 min, while previous studies with Cu-Oda and pUC18 showed similar activity but required a significantly longer exposure of 20 h (69). To probe the DNA damage of Cu_2_-BPL-C6 further, in-liquid AFM analysis with pUC19 revealed accelerated activity in the presence of ascorbate, while experiments in the absence of reductant show comparable cleavage activity to Cu-prodigiosin—a marine alkaloid with known ‘self-activated’ DNA damage potential (20)––but with substantially higher condensation effects mediated by the BPL-C6 complex.

The DNA damaging effects of Cu_2_-BPL-C6 extend to PBMCs that were employed to probe genomic DNA damaging activity of the complex. These effects are significant and produce an ∼6-fold increase compared to the untreated control. Several BER enzymes were probed in this analysis and, with Endo IV most efficiently recognizing DNA damage lesions, it appears Cu_2_-BPL-C6 mediates the formation of apurinic and apyrimidinic lesions. The formation of abasic lesions has been associated with the monomeric chemical nuclease Cu-(Phen)_2_, which abstracts the C1’ hydrogen atom from deoxyribose (71–73). The data presented here appear consistent with this work and point to a conserved mode of DNA cleavage by this chemotype. When cells were prophylactically treated with specific antioxidants, a significant decrease in DNA damage was found in the presence of the known superoxide scavenger, tiron. Therefore, a conserved mechanism of oxidative damage appears consistent both *in vitro* and *in cellulo*.

Finally, NCI-60 analysis of the BPL-C6 ligand reveals that it is cytotoxic to several cancer cell lines including non-small cell lung cancer (NCI-H23), colon cancers (COLO 205 and HCC-2998), melanoma (SK-MEL-2 and SK-MEL-5) and breast cancers (MDA-MB-468 and BT549). The ligand was then examined using the DTP COMPARE algorithm. Here, the LC_50_ activity profile of the compound appears consistent with known DNA damaging and topoisomerase poisoning agents such as actinomycin D, mitomycin C, mithramycin, docetaxel, doxorubicin and epirubicin. Additional head-to-head experiments with Cu(II)-free BPL-C6 and the active metal complex Cu_2_-BPL-C6 reveals differences on cell viability, particularly in the context of MDA-MB-438, where a significant sensitization to this cell line occurs in the presence of copper(II). Although these initial anticancer results are encouraging, further *in vitro* and *in vivo* experiments are required to identify whether BPL-C6 sequesters intracellular copper as part of its detectable anticancer mode of activity. In summary, we presented a click chemistry method for generating a new type of dinuclear copper complex with potent DNA binding and damaging activity. In the context of generating new metallodrugs, this synthetic route may prove valuable in generating new libraries of candidate metallodrugs that furnish enhanced DNA recognition and damaging properties.

## Supplementary Material

gkae1250_Supplemental_File

## Data Availability

Cambridge Crystallographic Data Centre CCDC deposition number 2332750 (https://www.ccdc.cam.ac.uk/). AFM data, including raw and processed image files and .csv files containing statistical analysis are available *via* ORDA (https://doi.org/10.15131/shef.data.27613857).
