# Supplementary material for: Design and in vitro anticancer assessment of a click chemistry-derived dinuclear copper artificial metallo-nuclease
Source: Nucleic Acids Res. 2025 Jan 7;53(1):gkae1250. doi: 10.1093/nar/gkae1250 (PMC11705080; doi:10.1093/nar/gkae1250)
Supplement: gkae1250_Supplemental_File [file gkae1250_supplemental_file.pdf]

Supplementary Information for:

Design and In Vitro Anticancer Assessment of a Click Chemistry-Derived Dinuclear Copper Artificial Metallo-Nuclease

Simon Poole,[1] Obed Akwasi Aning,[2] Vickie McKee,[1,3] Thomas Catley,[4] Aaraby Yoheswaran Nielsen,[5] Helge Thisgaard,[5,6] Pegah Johansson,[7,8] Georgia Menounou,[1] Joseph Hennessy,[1] Creina Slator,[1] Alex Gibney,[1] Alice Pyne,[4] Bríonna McGorman,[1†] Fredrik Westerlund,[2†] Andrew Kellett[1,*]

[1] School of Chemical Sciences, Dublin City University, Glasnevin, Dublin 9, Ireland.
[2] Department of Life Sciences, Chalmers University of Technology, Gothenburg, Sweden.
[3] Department of Physics, Chemistry and Pharmacy University of Southern Denmark Campusvej 55, 5230 Odense M, Denmark.
[4] Department of Materials Science and Engineering, University of Sheffield, Sheffield, UK.
[5] PET & Cyclotron Unit, Department of Nuclear Medicine, Odense University Hospital, Odense, Denmark.
[6] Department of Clinical Research, University of Southern Denmark, Odense, Denmark.
[7] Department of Clinical Chemistry, Sahlgrenska University Hospital, Region Vastra Gotaland, Gothenburg, Sweden.
[8] Department of Laboratory Medicine, Institute of Biomedicine, Sahlgrenska Academy at University of Gothenburg, Sweden.

* To whom correspondence should be addressed. Tel: +353 1 7005461; E-mail: andrew.kellett@dcu.ie
† Authors that contributed equally.

Table of Contents

S-1: METAL COMPLEXES WITH DISCRETE DRUG-DNA BINDING PROPERTIES.............................. 3

S-2: ^{1}H AND C^{13} NMR SPECTRA.. 4

S-3: IR SPECTRA.. 9

S-4: MASS SPECTRA...11

S-5: SINGLE CRYSTAL X-RAY DIFFRACTION...15

S-6: ELEMENTAL ANALYSIS 20

S-7: ETHIDIUM BROMIDE DISPLACEMENT EXPERIMENTS..21

S-8: NCI-60 GI50 AND TGI DATA 23

S-9: BARD DNA BINDING ANALYSIS..25

S-10: SELF-ACTIVATION CONTROL ELECTROPHORESIS DATA 26

S-11: ATOMIC FORCE MICROSCOPY 27

S-1: Metal complexes with discrete drug-DNA binding properties

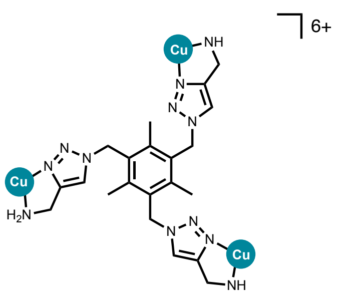

Cu-TC-1

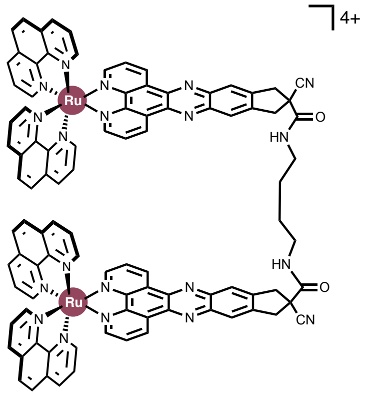

[μ-c4(cpdppz)$_2$(phen)$_4$Ru$_2$]$^{4+}$

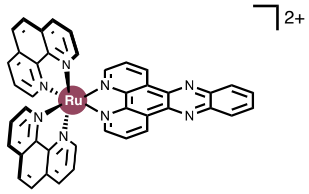

[Ru(phen)$_2$(dppz)]$^{2+}$

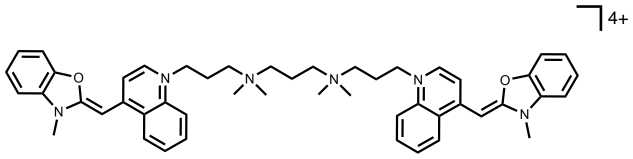

YOYO-1

Figure S1: Molecular structures of Cu-TC-1, [μ-c4(cpdppz)$_2$(phen)$_4$Ru$_2$]$^{4+}$, [Ru(phen)$_2$(dppz)]$^{2+}$ and YOYO-1.

S-2: ^{1}H and C^{13} NMR spectra

^{1}H NMR spectrum of 1,4-bis(1-(1,10-phenanthrolin-5-yl)-1H-1,2,3-triazol-4-yl)butane ***(3)***

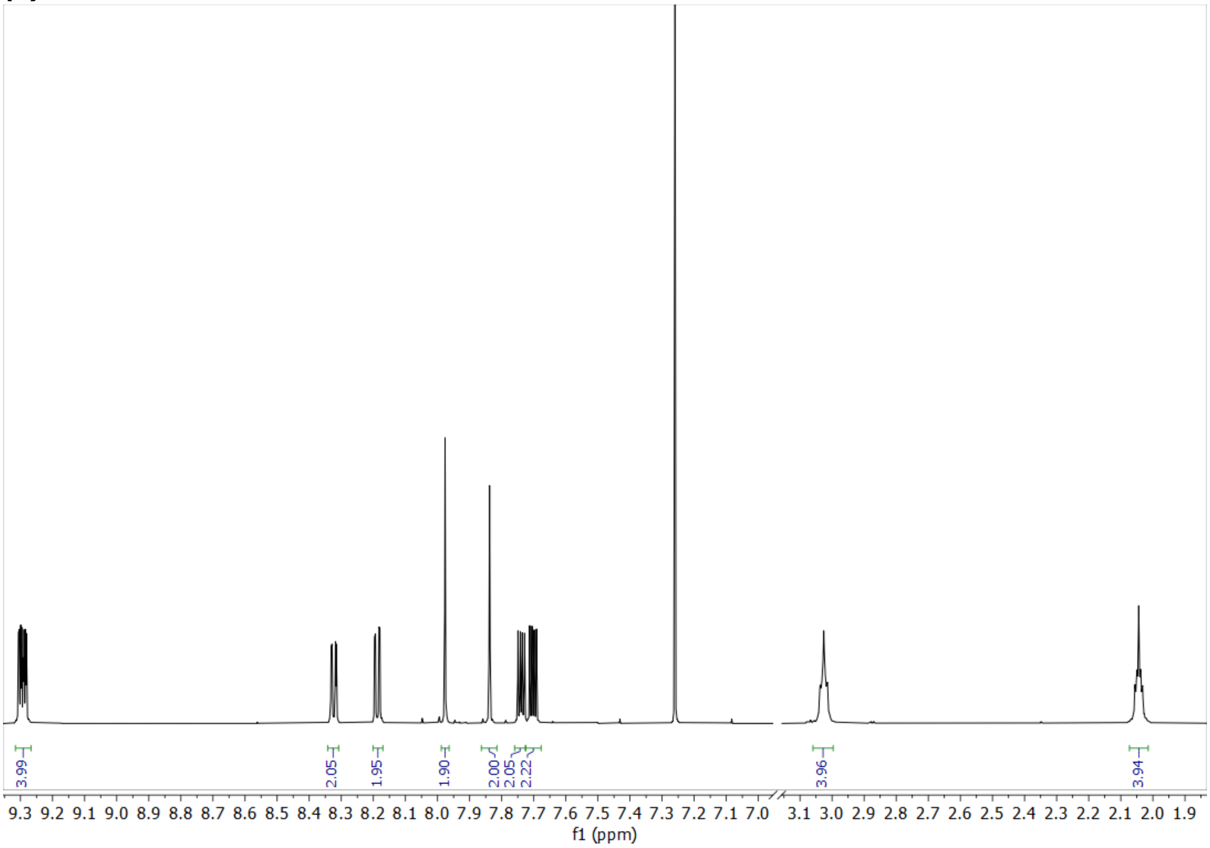

Figure S2: 1,4-bis(1-(1,10-phenanthrolin-5-yl)-1H-1,2,3-triazol-4-yl)butane ^{1}H NMR spectrum.

*C[13]-NMR spectrum of 1,4-bis(1-(1,10-phenanthrolin-5-yl)-1H-1,2,3-triazol-4-yl)butane (**3**)*

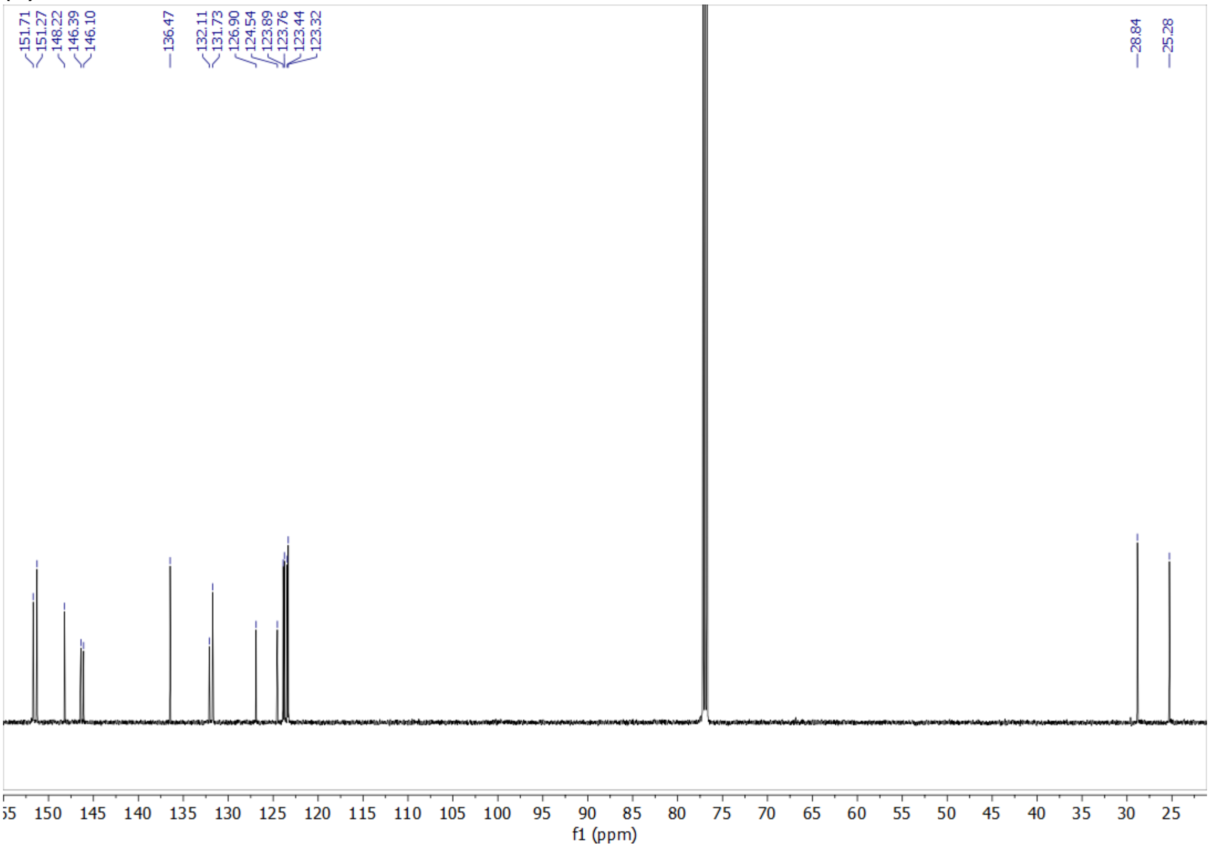

Figure S3: 1,4-bis(1-(1,10-phenanthrolin-5-yl)-1H-1,2,3-triazol-4-yl)butane C[13]-NMR spectrum.

*DEPT spectrum of 1,4-bis(1-(1,10-phenanthrolin-5-yl)-1H-1,2,3-triazol-4-yl)butane (**3**)*

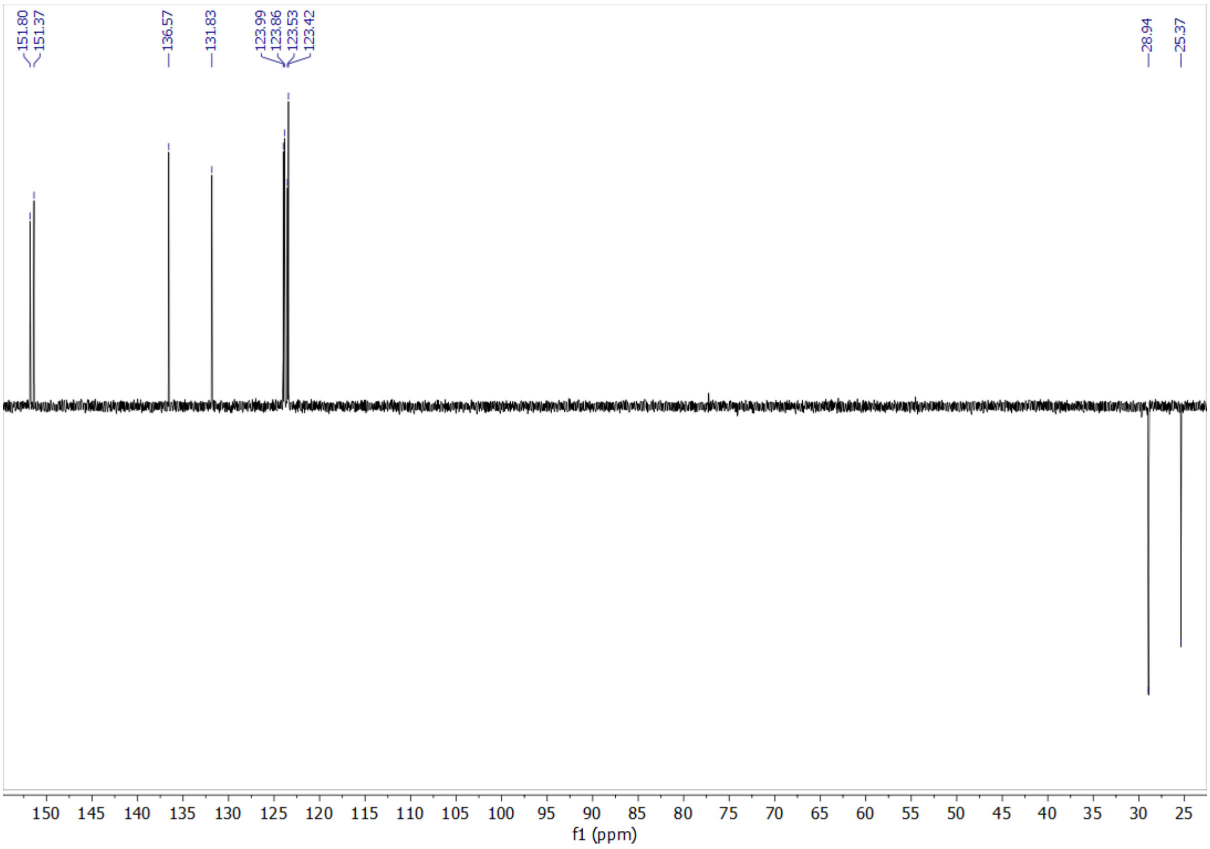

Figure S4: 1,4-bis(1-(1,10-phenanthrolin-5-yl)-1H-1,2,3-triazol-4-yl)butane (DEPT spectrum).

*^{1}H NMR spectrum of 5,6-epoxy-5,6-dihydro-1,10-phenanthroline (**1**)*

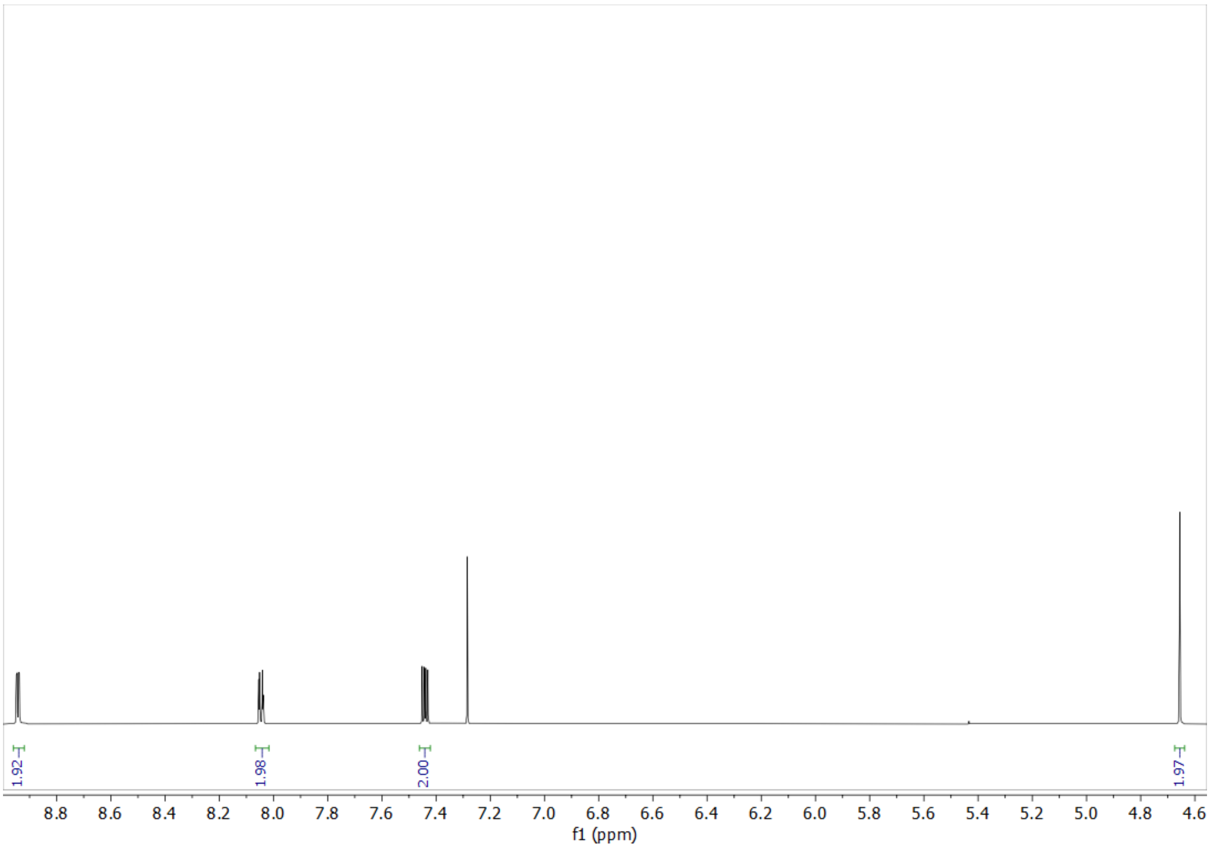

Figure S5: 5,6-epoxy-5,6-dihydro-1,10-phenanthroline ^{1}H NMR spectrum.

*^{1}H NMR spectrum of 5-azido-1,10-phenanthroline (**2**)*

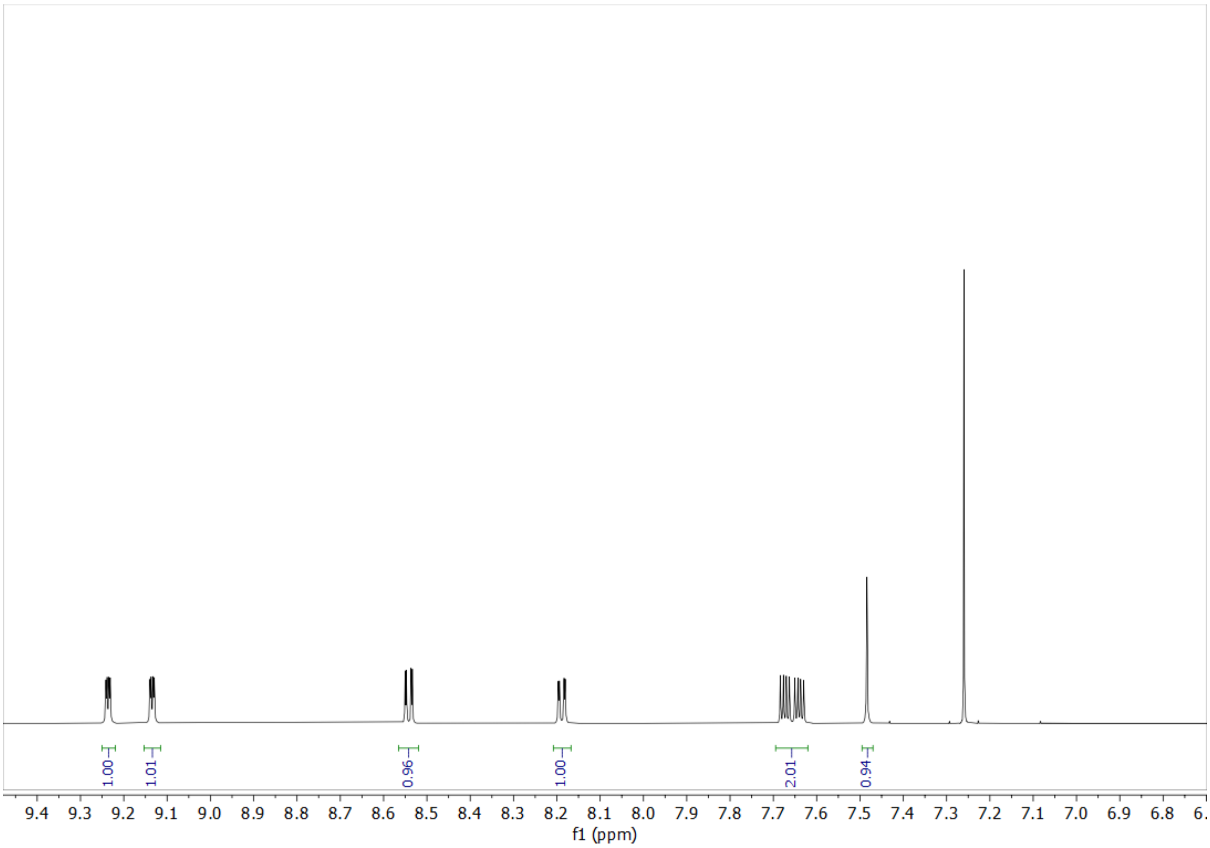

Figure S6: 5-azido-1,10-phenanthroline ^{1}H NMR spectrum.

S-3: IR Spectra

*IR spectrum of 1,4-bis(1-(1,10-phenanthrolin-5-yl)-1H-1,2,3-triazol-4-yl)butane (**3**)*

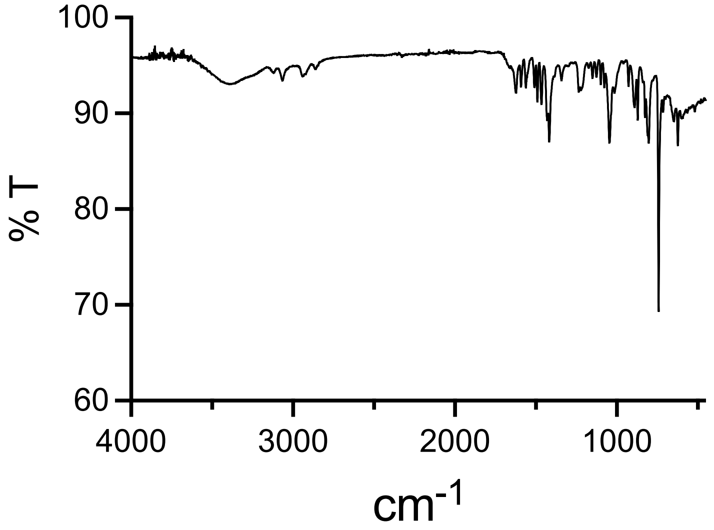

Figure S7: 1,4-bis(1-(1,10-phenanthrolin-5-yl)-1H-1,2,3-triazol-4-yl)butane IR spectrum.

*IR spectrum of 5,6-dihydro-5,6-epoxy-1,10-phenanthroline (**1**)*

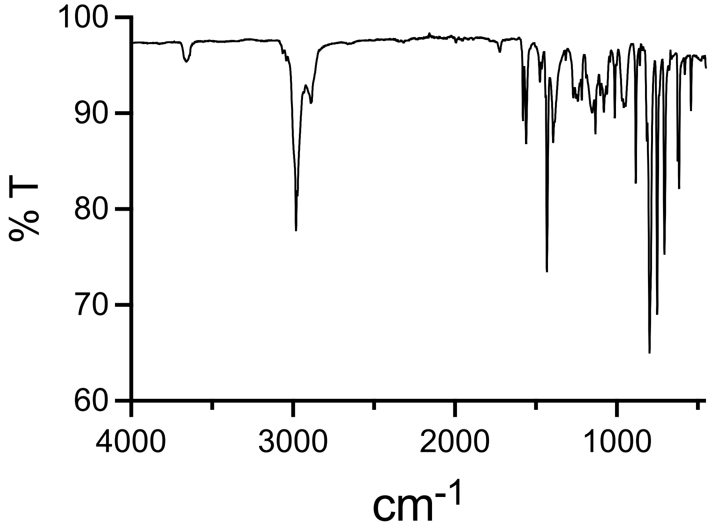

Figure S8: 5,6-epoxy-5,6-dihydro-1,10-phenanthroline IR spectrum.

*IR spectrum of 5-azido-1,10-phenanthroline (**2**)*

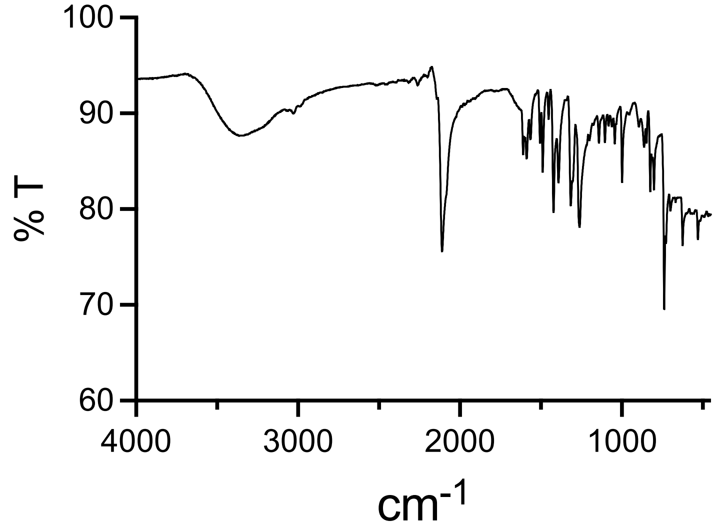

Figure S9: 5-azido-1,10-phenanthroline IR spectrum.

*IR spectrum of Cu_2-BPL-C6 (**4**)*

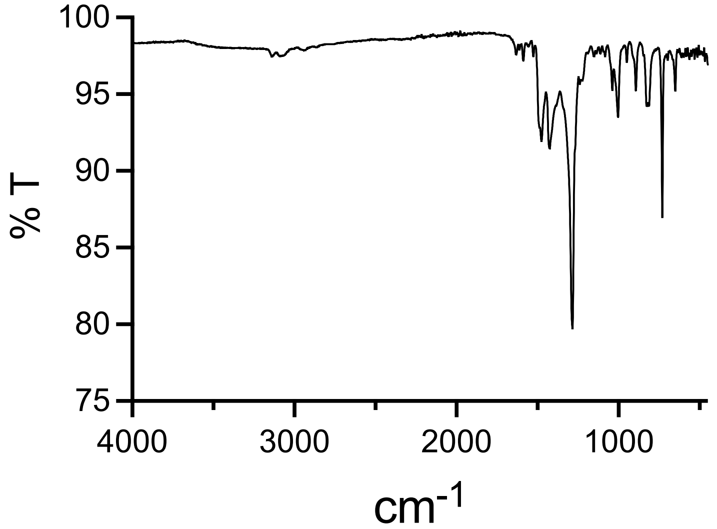

Figure S10: Cu_2-BPL-C6 IR spectrum.

S-4: Mass spectra

Mass Spectrum of Cu(II):BPL-C6 1:1 (In-situ)

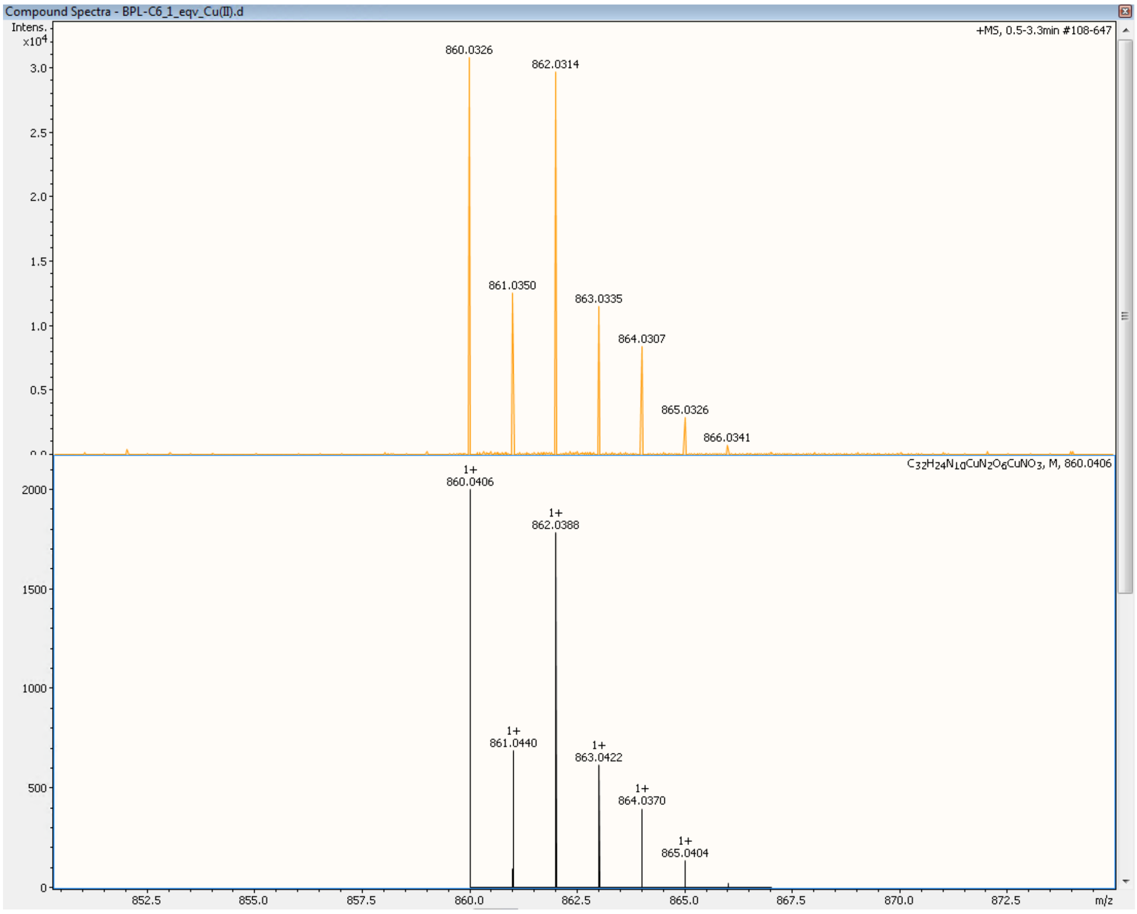

Figure S11: In-situ complex mass spectrum co-incubated with 1 eqv. of $Cu(NO_3)_2$, indicating the predicted mass spectrum on the bottom and the obtained mass spectrum on the top showing the formation of the 2:1 Cu(II):ligand complex $[(C_{32}H_{24})Cu_2(NO_3)_3]^+$.

Mass Spectrum of Cu(II):BPL-C6 2:1 (In-situ)

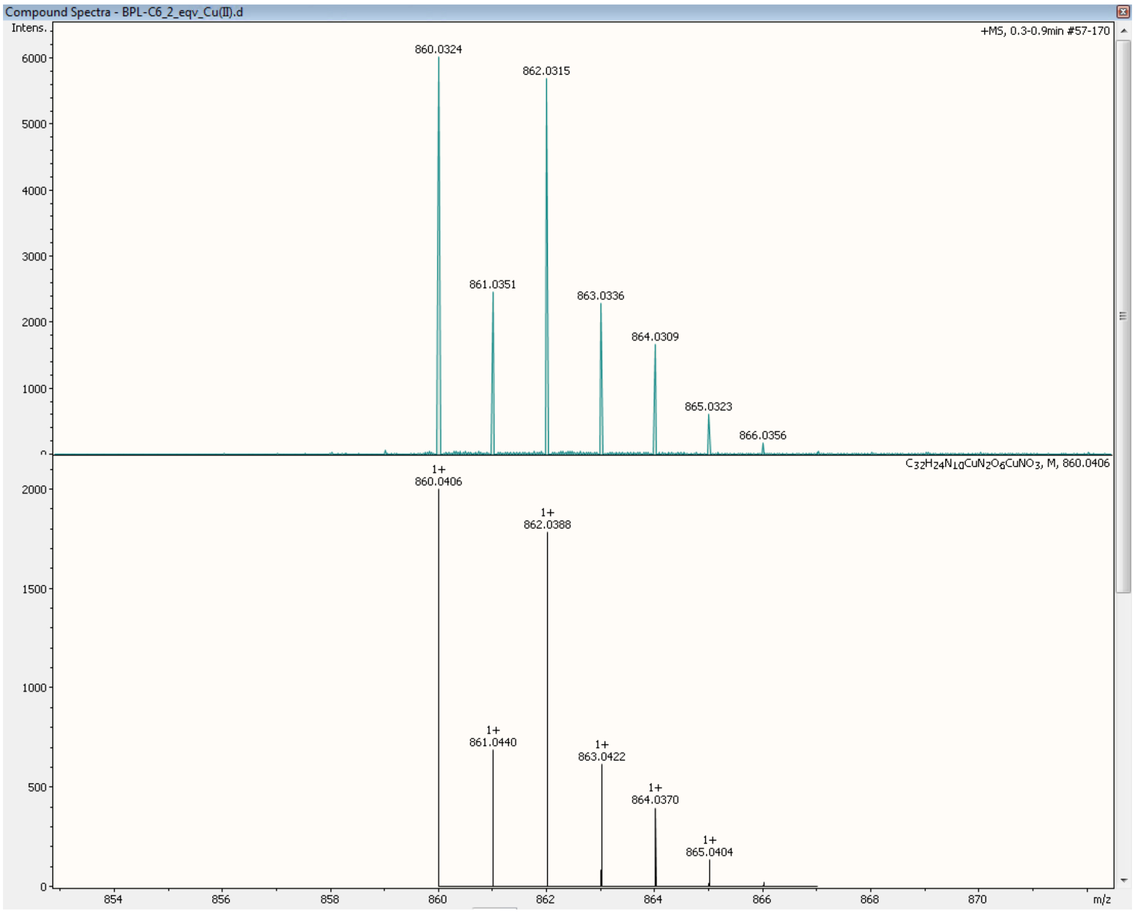

Figure S12: In-situ complex mass spectrum co-incubated with 2 eqv. of $Cu(NO_3)_2$, indicating the predicted mass spectrum on the bottom and the obtained mass spectrum on the top showing the formation of the 2:1 Cu(II):ligand complex $[(C_{32}H_{24})Cu_2(NO_3)_3]^+$.

Mass Spectrum of Cu(II):BPL-C6 10:1 (In-situ)

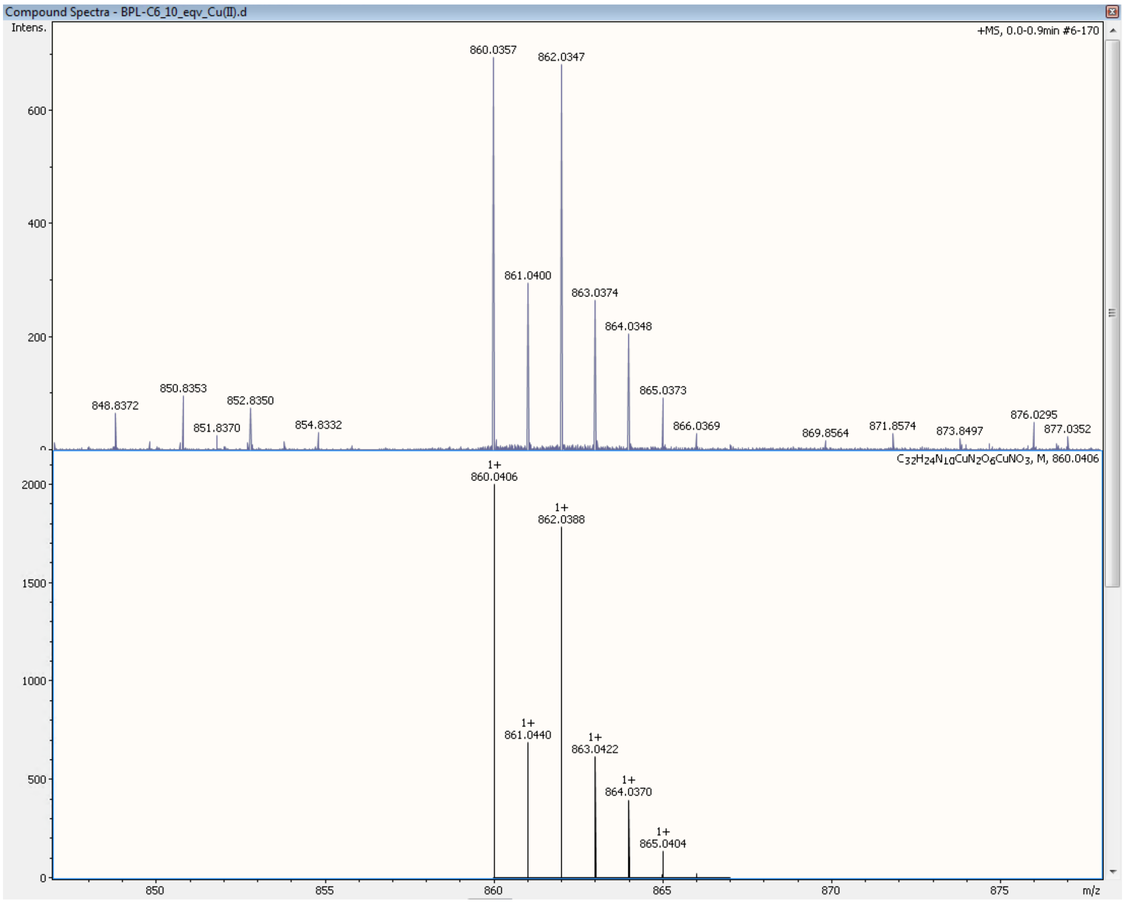

Figure S13: In-situ complex mass spectrum co-incubated with 10 eqv. of $Cu(NO_3)_2$, indicating the predicted mass spectrum on the bottom and the obtained mass spectrum on the top showing the formation of the 2:1 Cu(II):ligand complex $[(C_{32}H_{24})Cu_2(NO_3)_3]^+$.

*Mass spectrum of 1,4-bis(1-(1,10-phenanthrolin-5-yl)-1H-1,2,3-triazol-4-yl)butane (**3**)*

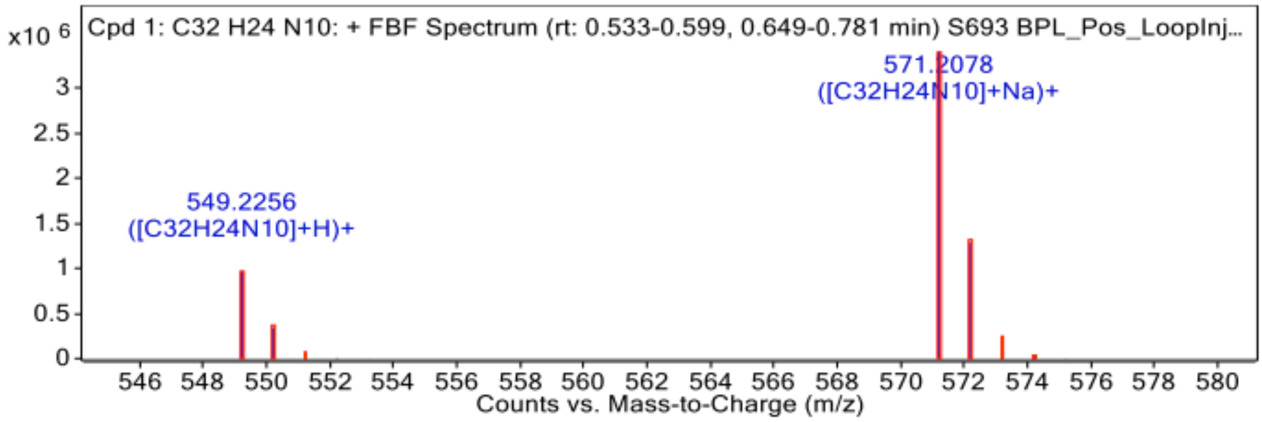

Figure S14: 1,4-bis(1-(1,10-phenanthrolin-5-yl)-1H-1,2,3-triazol-4-yl)butane mass spectrum.

*Mass Spectrum of Cu_2-BPL-C6 Discrete Complex (**4**)*

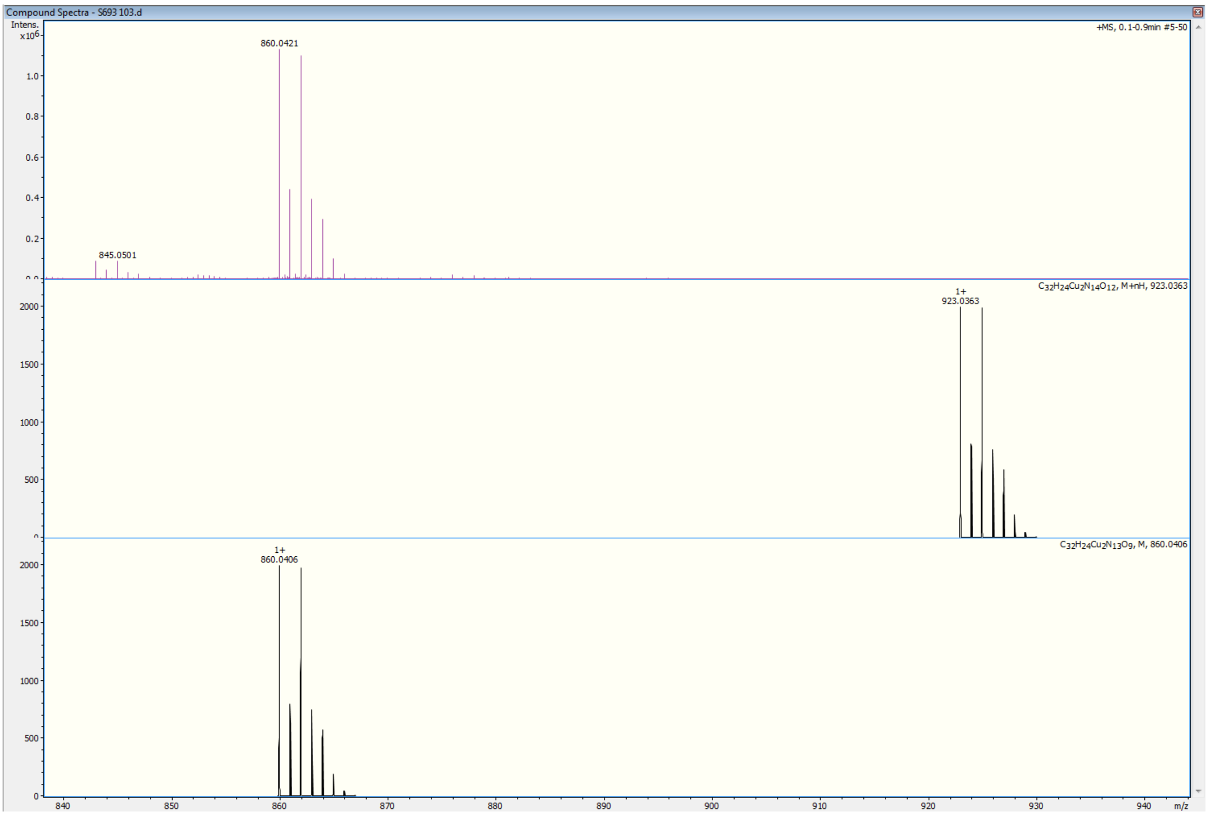

Figure S15: Cu_2-BPL-C6 discrete complex mass spectrum indicating the predicted mass spectrum on the bottom and the obtained mass spectrum on the top showing the formation of the 2:1 Cu(II):ligand complex $[(C_{32}H_{24})Cu_2(NO_3)_3]^+$.

S-5: Single crystal X-ray diffraction

BPL-C6 (**3**) $C_{32}H_{24}N_{10}\cdot 2(CHCl_3)$

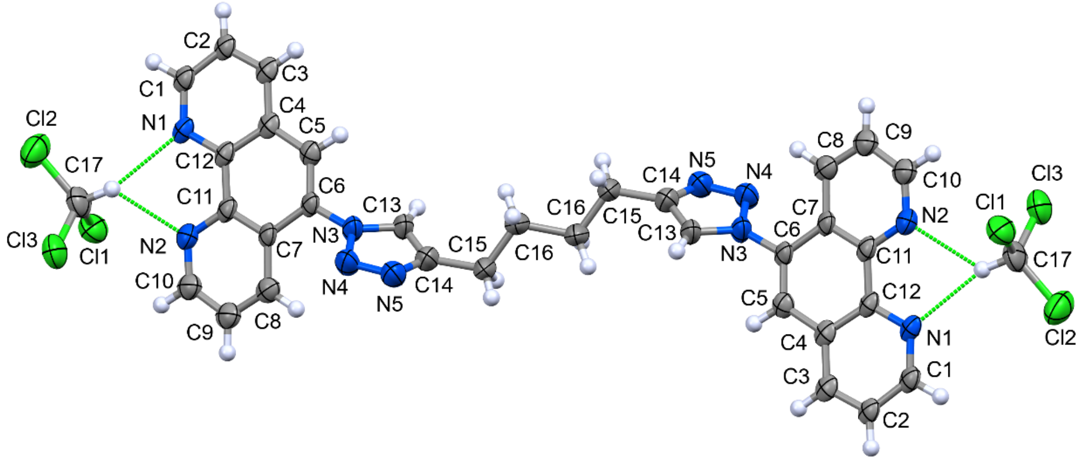

Figure S16: Labelled, 50% probability ellipsoid plot of the centrosymmetric molecule (disorder omitted).

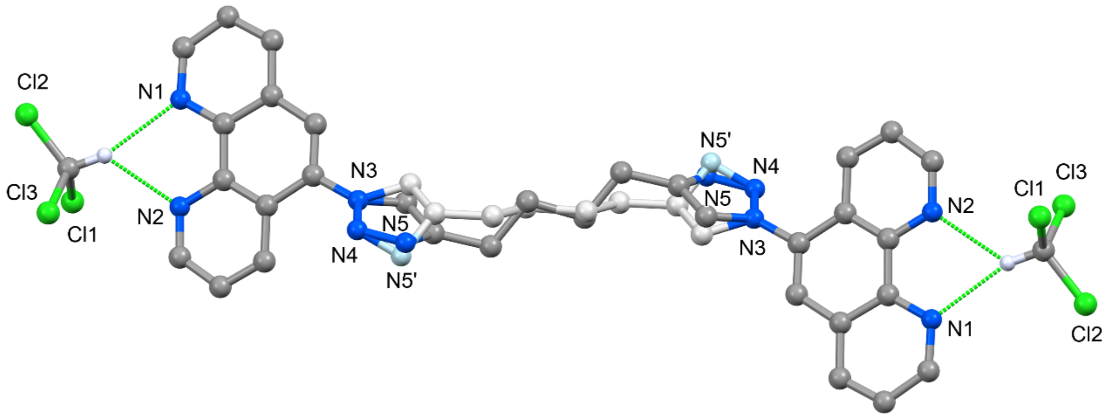

Figure S17: Perspective view showing disorder.

The crystals were small and of poor quality and hence the structure refinement is of relatively low precision, however, the main features of the structure are clear. The molecule lies on a centre of symmetry, though the saturated chain and 5-membered rings are disordered. The disorder was modelled with 50% occupancy of two overlapping orientations. The chloroform solvate molecules are hydrogen bonded to the nitrogen atoms of the phen group (C-H····Cl distances 3.422(17) and 3.079(16))Å to N1 and N2, respectively).

The BPL-C6 molecules are packed in layers with significant π-stacking of the phen groups.

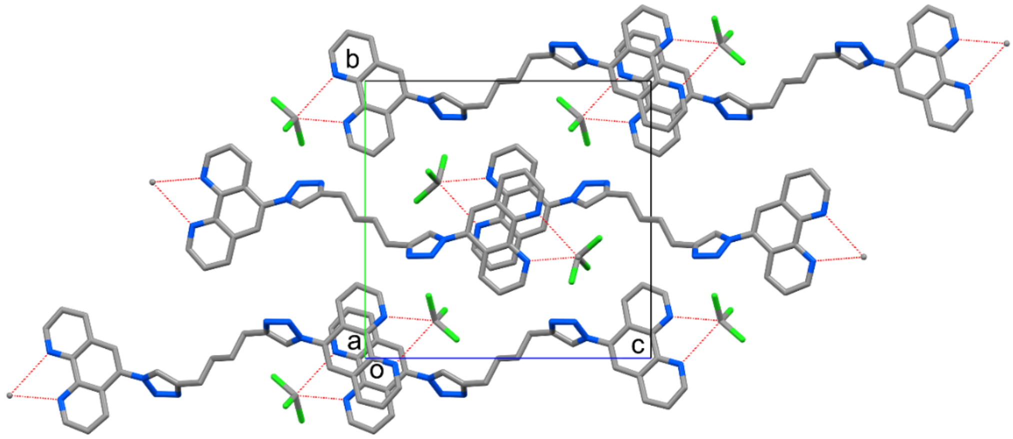

Figure S18: Packing diagram viewed down the a-axis showing π-stacking. Disorder omitted; H-bonds shown as dashed red lines.

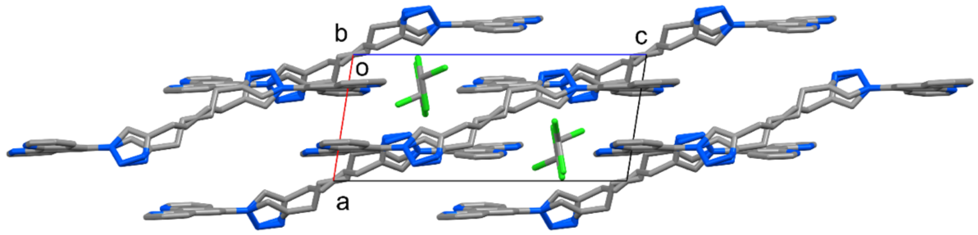

Figure S19: Packing diagram viewed down the b axis showing layers of BPL-C6 molecules.

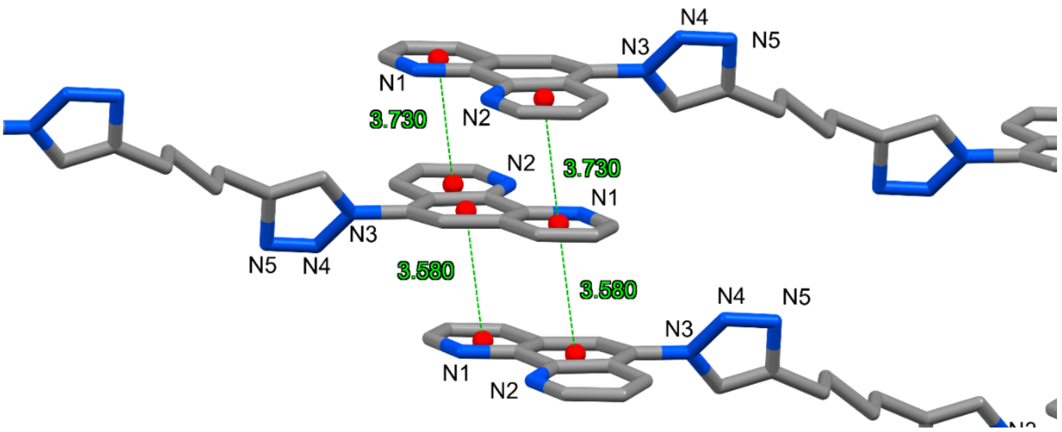

Figure S20: Detail of the π-stacking. Ring centroids shown as red spheres, centroid-centroid distances given in Å.

Experimental

The data were collected at 100(1)K on a Synergy, Dualflex, AtlasS2 diffractometer using Cu*K*α radiation (λ = 1.54184 Å) and the *CrysAlis PRO* 1.171.42.49 suite[1]. The crystal was of very poor quality, small and layered with very streaky reflections in one direction. The data were initially processed as a triplet (ratios 41:20:25) and the structure solved

using data from the first component. Subsequently, data processed as a single crystal (40% of the reflections) were used as including twinning showed no improvement to the refinement. Using shelXle[2] and Olex2[3] the structure was solved by dual space methods (SHELXT[4]) and refined on F^2 using all the reflections (SHELXL-2019/2[5]). All the non-hydrogen atoms were refined using anisotropic atomic displacement parameters and hydrogen atoms were inserted at calculated positions using a riding model. Crystal parameters, data collection and structure refinement details are summarised in Table S1.

Table S1: Crystal data and structure refinement for exp_1165_BPLC6.

Identification code	bplc6
Empirical formula	$C_{34}H_{26}N_{10}Cl_6$
Formula weight	787.35
Temperature/K	100.00(10)
Crystal system	monoclinic
Space group	$P2_1/n$
a/Å	6.9059(14)
b/Å	15.297(3)
c/Å	16.276(3)
α/°	90
β/°	98.35(2)
γ/°	90
Volume/Å^3	1701.1(6)
Z	2
ρ_{calc}g/cm^3	1.537
μ/mm^{-1}	4.967
F(000)	804.0
Crystal size/mm^3	0.113 × 0.027 × 0.012
Radiation	Cu Kα (λ = 1.54184)
2θ range for data collection/°	7.972 to 101.968
Index ranges	-6 ≤ h ≤ 6, -15 ≤ k ≤ 15, -16 ≤ l ≤ 10
Reflections collected	3329
Independent reflections	1769 [R_{int} = 0.0820, R_{sigma} = 0.1401]
Data/restraints/parameters	1769/524/271
Goodness-of-fit on F^2	1.046
Final R indexes [I>=2σ (I)]	R_1 = 0.1098, wR_2 = 0.2729
Final R indexes [all data]	R_1 = 0.1703, wR_2 = 0.3246
Largest diff. peak/hole / e Å^{-3}	0.70/-0.41
CCDC Deposition number	2332750

Geometric parameters (Å, º) for exp_1165_bplc6

N1—C1	1.319 (15)	N3—C13	1.351 (16)
N1—C12	1.355 (13)	N3—C13'	1.39 (2)
N2—C10	1.306 (16)	N4—N5	1.328 (14)
N2—C11	1.343 (15)	N4—N5'	1.31 (2)
C1—C2	1.371 (16)	N5—C14	1.42 (2)
C2—C3	1.358 (15)	C13—C14	1.330 (19)
C3—C4	1.393 (17)	C14—C15	1.50 (2)
C4—C5	1.432 (15)	C15—C16	1.55 (2)
C4—C12	1.440 (17)	C16—C16^{i}	1.55 (4)
C5—C6	1.351 (15)	N5'—C14'	1.43 (3)
C6—C7	1.430 (16)	C13'—C14'	1.34 (3)
C6—N3	1.414 (14)	C14'—C15'	1.50 (2)
C7—C8	1.430 (16)	C15'—C16'	1.56 (3)
C7—C11	1.417 (15)	C16'—C16'^{i}	1.66 (12)
C8—C9	1.375 (17)	Cl1—C17	1.750 (14)
C9—C10	1.392 (17)	Cl2—C17	1.758 (14)
C11—C12	1.443 (18)	Cl3—C17	1.746 (15)
N3—N4	1.320 (13)		
C1—N1—C12	117.1 (11)	N4—N3—C13	110.8 (11)
C10—N2—C11	118.8 (11)	N4—N3—C13'	101.6 (15)
N1—C1—C2	125.8 (12)	C13—N3—C6	125.3 (11)
C3—C2—C1	118.6 (13)	C13'—N3—C6	130.4 (16)
C2—C3—C4	119.2 (12)	N3—N4—N5	108.2 (11)
C3—C4—C5	123.2 (11)	N5'—N4—N3	112.1 (19)
C3—C4—C12	118.4 (11)	N4—N5—C14	104.9 (13)
C5—C4—C12	118.2 (11)	C14—C13—N3	105.6 (14)
C6—C5—C4	121.8 (11)	N5—C14—C15	122.0 (15)
C5—C6—C7	121.3 (11)	C13—C14—N5	108.3 (13)
C5—C6—N3	120.2 (11)	C13—C14—C15	129.5 (16)
N3—C6—C7	118.4 (10)	C14—C15—C16	111.8 (14)
C6—C7—C8	123.7 (11)	C16^{i}—C16—C15	109 (2)
C11—C7—C6	119.5 (11)	N4—N5'—C14'	97 (2)
C11—C7—C8	116.7 (11)	C14'—C13'—N3	107 (2)
C9—C8—C7	118.6 (12)	N5'—C14'—C15'	115 (2)
C8—C9—C10	119.4 (14)	C13'—C14'—N5'	106 (2)

N2—C10—C9	123.6 (13)	C13'—C14'—C15'	134 (3)
N2—C11—C7	122.9 (12)	C14'—C15'—C16'	110 (3)
N2—C11—C12	117.9 (10)	C15'—C16'—C16'^{ii}	108 (6)
C7—C11—C12	119.2 (11)	Cl1—C17—Cl2	111.6 (7)
N1—C12—C4	120.8 (12)	Cl3—C17—Cl1	110.7 (8)
N1—C12—C11	119.5 (11)	Cl3—C17—Cl2	111.1 (8)
C4—C12—C11	119.7 (10)		
N4—N3—C6	123.9 (9)		

Hydrogen-bond geometry (Å, º) for bplc6

D—H···*A*	*D*—H	H···*A*	*D*···*A*	*D*—H···*A*
C17—H17···N1	1.00	2.47	3.422 (17)	159
C17—H17···N2	1.00	2.36	3.079 (16)	128

Symmetry code: (ii) -*x*+2, -*y*+1, -*z*+1.

S-6: Elemental analysis

*Elemental Analysis of Cu_2-BPL-C6$(NO_3)_4$.3H_2O (**4**)*

Results

CHN Elemental Analysis

Element	Nitrogen %	Carbon %	Hydrogen %
Theoretical Results (filled by student)	21.23	41.61	2.62
Experimental results **Run 1**	20.42	39.56	2.88
Experimental results **Run 2**	20.06	38.83	2.81

Figure S21: Elemental analysis performed on Cu_2-BPL-C6$(NO_3)_4$.3H_2O.

S-7: Ethidium bromide displacement experiments

DNA binding investigation with first row transition metals and Cu_2-BPL-C6

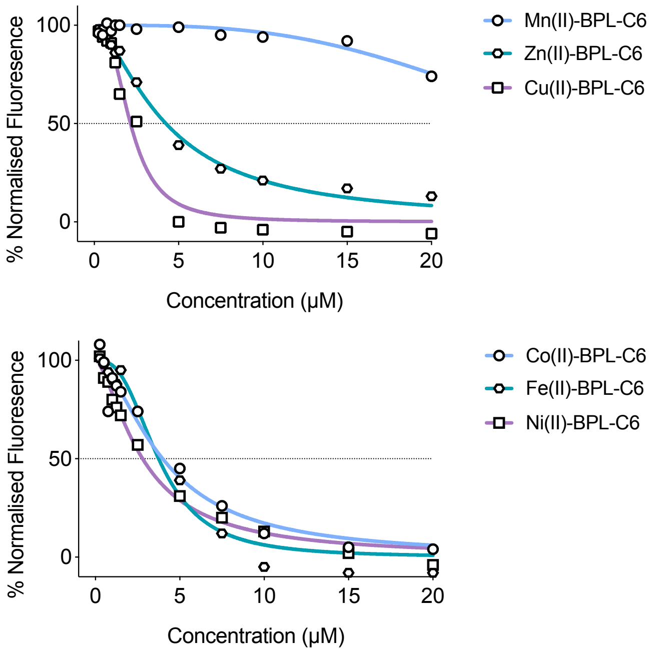

Figure S22: Preliminary DNA binding profile (single point analysis) of first row transition metals in the presence of BPL-C6 for 1 h with calf-thymus DNA using fluorescence quenching of EtBr, showing preference for Cu_2-BPL-C6 as the lead candidate.

DNA binding control experiment showing the influence of BPL-C6 and Cu_2-BPL-C6 on EtBr fluorescence

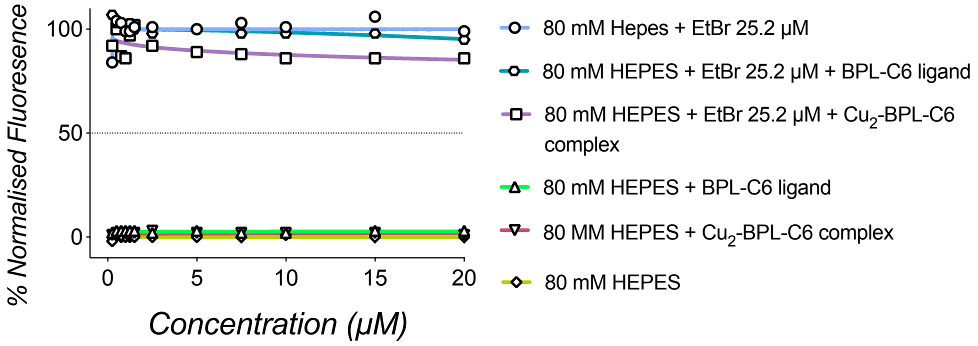

Figure S23: Fluorescence quenching control experiments (single point analysis) showing the influence of BPL-C6 and Cu_2-BPL-C6 on the emission of EtBr using 80 mM HEPES as a blank.

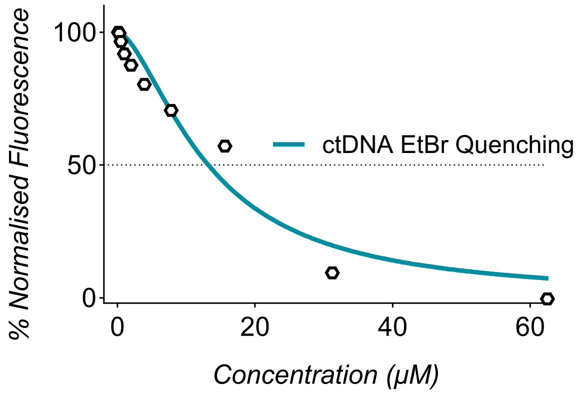

Figure S24: Fluorescence quenching control experiments indicating fluorescence depletion upon Cu_2-BPL-C6 incubation with DNA.

S-8: NCI-60 GI50 and TGI data

NCI-60 GI_{50} and TGI data

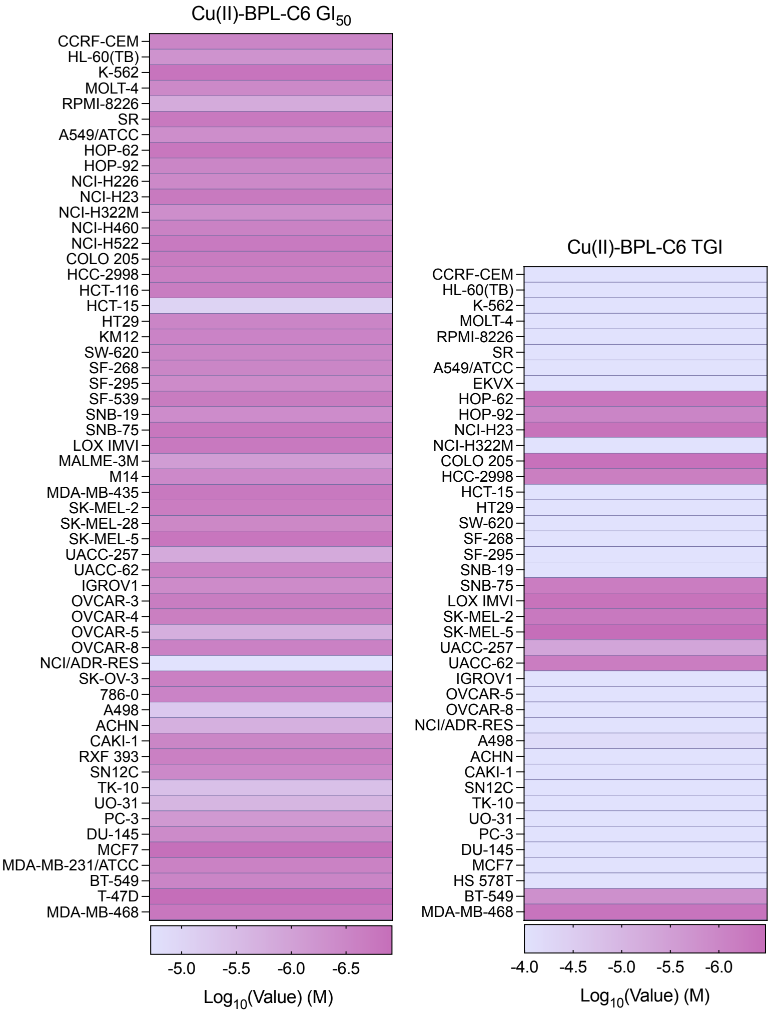

Figure S25: NCI-60 data for the interaction of Cu(II)-free BPL-C6 with cancer cell lines monitoring GI_{50} (left) and TGI (right).

Panel/Cell Line	Time Zero	Ctrl	Mean Optical Densities (Log(Concentration)) -8.0	-7.0	-6.0	-5.0	-4.0	Percent Growth (Log(Concentration)) -8.0	-7.0	-6.0	-5.0	-4.0	GI50	TGI	LC50
**Leukemia**															
CCRF-CEM	0.517	2.428	2.369	2.084	0.871	0.670	0.841	97	82	19	8	17	3.19E-7	> 1.00E-4	> 1.00E-4
HL-60(TB)	0.600	2.584	2.511	2.600	1.210	0.866	1.103	96	101	31	13	25	5.31E-7	> 1.00E-4	> 1.00E-4
K-562	0.212	2.184	2.015	1.370	0.524	0.407	0.555	91	59	16	10	17	1.59E-7	> 1.00E-4	> 1.00E-4
MOLT-4	0.451	2.293	2.379	2.067	0.788	0.569	0.993	105	88	18	6	29	3.49E-7	> 1.00E-4	> 1.00E-4
RPMI-8226	0.732	2.445	2.457	2.278	1.748	0.889	1.013	101	90	59	9	16	1.53E-6	> 1.00E-4	> 1.00E-4
SR	0.605	2.537	2.412	1.849	0.818	0.852	1.293	94	64	11	13	36	1.86E-7	> 1.00E-4	> 1.00E-4
**Non-Small Cell Lung Cancer**															
A549/ATCC	0.313	2.312	2.376	2.299	0.752	0.500	0.874	103	99	22	9	28	4.34E-7	> 1.00E-4	> 1.00E-4
EKVX	0.849	2.378	2.358	2.263	1.900	1.154	1.649	99	92	69	20	52	.	> 1.00E-4	> 1.00E-4
HOP-62	0.895	2.322	2.194	2.046	0.544	0.553	0.652	91	81	-39	-38	-27	1.80E-7	4.71E-7	> 1.00E-4
HOP-92	1.140	1.545	1.551	1.568	1.107	1.020	1.082	101	106	-3	-11	-5	3.25E-7	9.40E-7	> 1.00E-4
NCI-H226	0.899	1.870	1.842	1.683	1.152	0.702	1.364	97	81	26	-22	48	3.64E-7	.	> 1.00E-4
NCI-H23	0.639	2.057	1.963	1.896	0.321	0.297	0.289	93	89	-50	-54	-55	1.90E-7	4.37E-7	1.15E-6
NCI-H322M	0.785	2.378	2.354	2.472	1.089	0.933	1.258	99	106	19	9	30	4.40E-7	> 1.00E-4	> 1.00E-4
NCI-H460	0.302	2.658	2.697	2.477	0.300	0.607	0.762	102	92	0	13	20	2.85E-7	.	> 1.00E-4
NCI-H522	1.442	3.278	3.258	3.030	0.910	0.963	1.588	99	87	-37	-33	8	1.98E-7	.	> 1.00E-4
**Colon Cancer**															
COLO 205	0.714	2.296	2.414	2.322	0.248	0.182	0.332	107	102	-65	-75	-54	2.04E-7	4.06E-7	8.09E-7
HCC-2998	0.978	3.169	3.183	3.027	0.822	0.373	0.382	101	93	-16	-62	-61	2.50E-7	7.14E-7	5.50E-6
HCT-116	0.341	2.583	2.811	2.526	0.187	0.287	0.544	110	97	-45	-16	9	2.15E-7	.	> 1.00E-4
HCT-15	0.333	2.620	2.473	2.296	2.145	1.452	0.972	94	86	79	49	28	9.22E-6	> 1.00E-4	> 1.00E-4
HT29	0.260	1.901	2.022	1.697	0.452	0.343	0.505	107	88	12	5	15	3.13E-7	> 1.00E-4	> 1.00E-4
KM12	0.590	2.436	2.532	2.035	0.900	0.493	0.806	105	78	17	-17	12	2.88E-7	.	> 1.00E-4
SW-620	0.342	2.122	1.933	1.795	0.644	0.425	1.197	89	82	17	5	48	3.08E-7	> 1.00E-4	> 1.00E-4
**CNS Cancer**															
SF-268	0.855	2.377	2.568	2.038	1.220	1.008	1.290	113	78	24	10	29	3.28E-7	> 1.00E-4	> 1.00E-4
SF-295	0.900	2.967	2.752	2.635	1.484	0.952	0.995	90	84	28	2	5	4.07E-7	> 1.00E-4	> 1.00E-4
SF-539	0.649	2.422	2.283	2.255	0.386	0.508	0.825	92	91	-41	-22	10	2.04E-7	.	> 1.00E-4
SNB-19	0.664	2.561	2.328	2.506	1.074	0.845	0.909	88	97	22	10	13	4.21E-7	> 1.00E-4	> 1.00E-4
SNB-75	0.911	1.394	1.393	1.258	0.770	0.204	0.733	100	72	-16	-78	-20	1.77E-7	6.64E-7	.
U251	0.243	1.460	1.405	1.282	0.220	0.367	0.887	96	85	-9	10	53	.	.	> 1.00E-4
**Melanoma**															
LOX IMVI	0.506	2.959	2.849	2.650	0.242	0.494	0.506	96	87	-52	-2	0	1.85E-7	4.22E-7	.
MALME-3M	0.576	1.088	1.080	1.077	0.810	0.110	0.734	98	98	46	-81	31	8.28E-7	.	.
M14	0.541	2.120	2.114	2.031	0.808	0.306	0.754	100	94	17	-44	13	3.74E-7	.	> 1.00E-4
MDA-MB-435	0.630	2.198	2.149	2.044	0.249	0.356	0.917	97	90	-61	-43	18	1.85E-7	.	.
SK-MEL-2	1.280	2.517	2.488	2.438	0.861	0.156	0.044	98	94	-33	-88	-97	2.21E-7	5.51E-7	2.06E-6
SK-MEL-28	0.641	2.208	2.143	2.036	0.883	0.621	0.898	96	89	15	-3	16	3.39E-7	.	> 1.00E-4
SK-MEL-5	0.933	3.248	3.127	2.948	0.186	0.271	0.050	95	87	-80	-71	-95	1.67E-7	3.32E-7	6.60E-7
UACC-257	1.011	2.729	2.649	2.631	2.161	0.646	0.907	95	94	67	-36	-10	1.46E-6	4.46E-6	> 1.00E-4
UACC-62	0.846	2.997	3.046	3.038	0.638	0.462	0.606	102	102	-25	-45	-28	2.57E-7	6.39E-7	> 1.00E-4
**Ovarian Cancer**															
IGROV1	0.511	2.299	2.350	2.682	0.575	1.007	0.971	103	121	4	28	26	4.04E-7	> 1.00E-4	> 1.00E-4
OVCAR-3	0.495	1.758	1.783	1.420	0.538	0.464	0.894	102	73	3	-6	32	2.15E-7	.	> 1.00E-4
OVCAR-4	0.875	2.046	1.956	1.842	0.828	0.808	1.415	92	83	-5	-8	46	2.34E-7	.	> 1.00E-4
OVCAR-5	0.691	2.539	2.567	2.418	1.824	1.013	1.606	102	93	61	17	49	1.81E-6	> 1.00E-4	> 1.00E-4
OVCAR-8	0.419	2.304	2.259	1.989	0.501	0.451	0.681	98	83	4	2	14	2.64E-7	> 1.00E-4	> 1.00E-4
NCI/ADR-RES	0.527	1.952	1.957	1.869	1.826	1.347	0.968	100	94	91	58	31	1.92E-5	> 1.00E-4	> 1.00E-4
SK-OV-3	0.803	1.632	1.656	1.613	0.569	0.690	1.083	103	98	-29	-14	34	2.37E-7	.	> 1.00E-4
**Renal Cancer**															
786-0	0.761	2.883	2.795	2.726	0.797	0.737	1.072	96	93	2	-3	15	2.94E-7	.	> 1.00E-4
A498	1.228	2.436	2.355	2.270	2.359	1.666	1.142	93	86	94	36	-7	5.75E-6	6.89E-5	> 1.00E-4
ACHN	0.295	1.561	1.545	1.478	1.082	0.511	0.895	99	93	62	17	47	1.86E-6	> 1.00E-4	> 1.00E-4
CAKI-1	0.802	2.470	2.184	2.295	1.018	0.881	1.066	83	89	13	5	16	3.28E-7	> 1.00E-4	> 1.00E-4
RXF 393	0.864	1.701	1.651	1.613	0.786	0.323	1.165	94	89	-9	-63	36	2.52E-7	.	.
SN12C	0.613	2.816	2.695	2.512	1.117	0.878	1.525	95	86	23	12	41	3.73E-7	> 1.00E-4	> 1.00E-4
TK-10	1.357	2.238	2.205	2.157	2.239	1.474	1.649	96	91	100	13	33	3.77E-6	> 1.00E-4	> 1.00E-4
UO-31	0.493	1.984	1.681	1.635	1.630	0.587	1.041	80	77	76	6	37	2.37E-6	> 1.00E-4	> 1.00E-4
**Prostate Cancer**															
PC-3	0.613	1.816	1.751	1.656	1.135	0.762	0.959	95	87	43	12	29	7.04E-7	> 1.00E-4	> 1.00E-4
DU-145	0.406	1.784	1.873	1.747	0.628	0.628	0.920	106	97	16	16	37	3.82E-7	> 1.00E-4	> 1.00E-4
**Breast Cancer**															
MCF7	0.509	2.447	2.287	1.613	0.513	0.623	0.902	92	57	0	6	20	1.32E-7	> 1.00E-4	> 1.00E-4
MDA-MB-231/ATCC	0.522	1.359	1.415	1.162	0.673	0.372	0.907	107	76	18	-29	46	2.84E-7	.	> 1.00E-4
HS 578T	0.945	2.388	2.161	2.244	1.254	1.240	1.705	84	90	21	20	53	.	> 1.00E-4	> 1.00E-4
BT-549	1.295	2.533	2.499	2.303	1.493	0.427	0.292	97	81	16	-67	-77	3.02E-7	1.56E-6	6.24E-6
T-47D	0.533	1.109	0.997	0.865	0.329	0.455	0.785	80	58	-38	-15	44	1.20E-7	.	> 1.00E-4
MDA-MB-468	0.726	1.490	1.483	1.335	0.429	0.302	0.362	99	80	-41	-58	-50	1.76E-7	4.58E-7	3.29E-6

Figure S26: NCI-60 raw data for the interaction of Cu(II)-free BPL-C6 with cancer cell lines

S-9: Bard DNA binding analysis

Fluorescent melting Bard Analysis

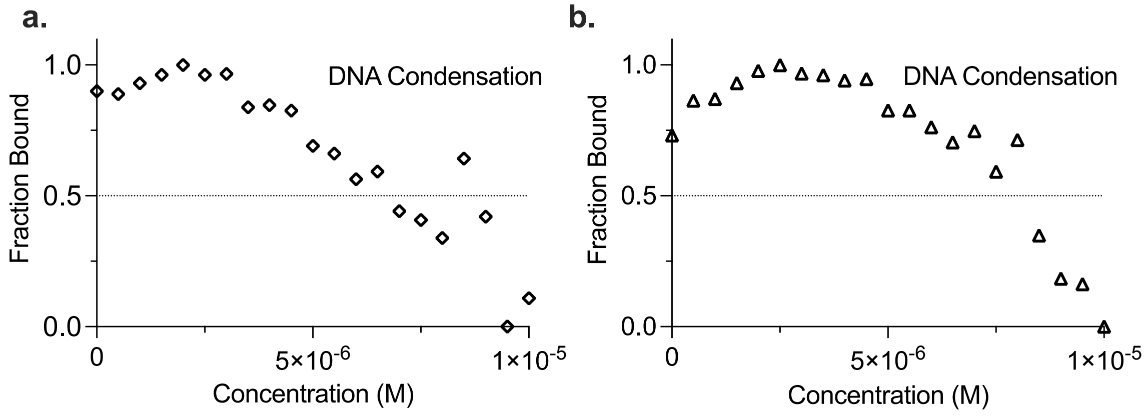

Figure S27: Bard analysis of FRET labelled DNA hairpins **a.** FRET-3 and **b.** FRET-4 at increasing concentrations of Cu_2-BPL-C6.

S-10: Self-activation control electrophoresis data

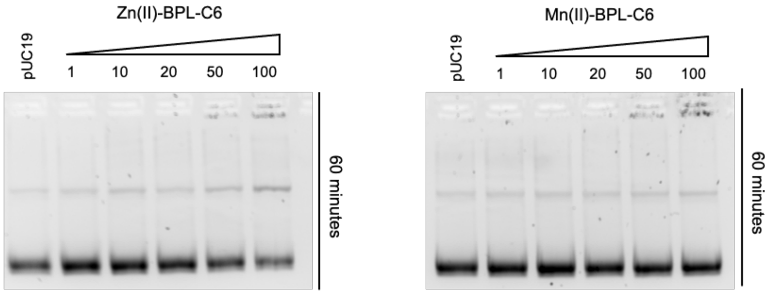

Figure S28: Self-activated pUC19 DNA cleavage investigation of first row transition metals (Zn(II) and Mn(II)) in the presence of BPL-C6 incubated for 1 h at 37°C.

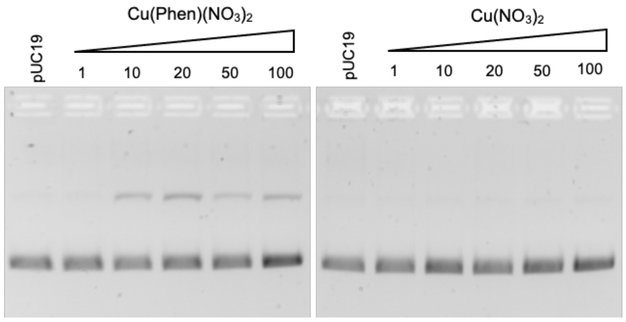

Figure S29: Self-activated pUC19 DNA cleavage investigation of $Cu(Phen)(NO_3)_2$ and $Cu(NO_3)_2$ incubated for 1 h at 37°C.

S-11: Atomic Force Microscopy

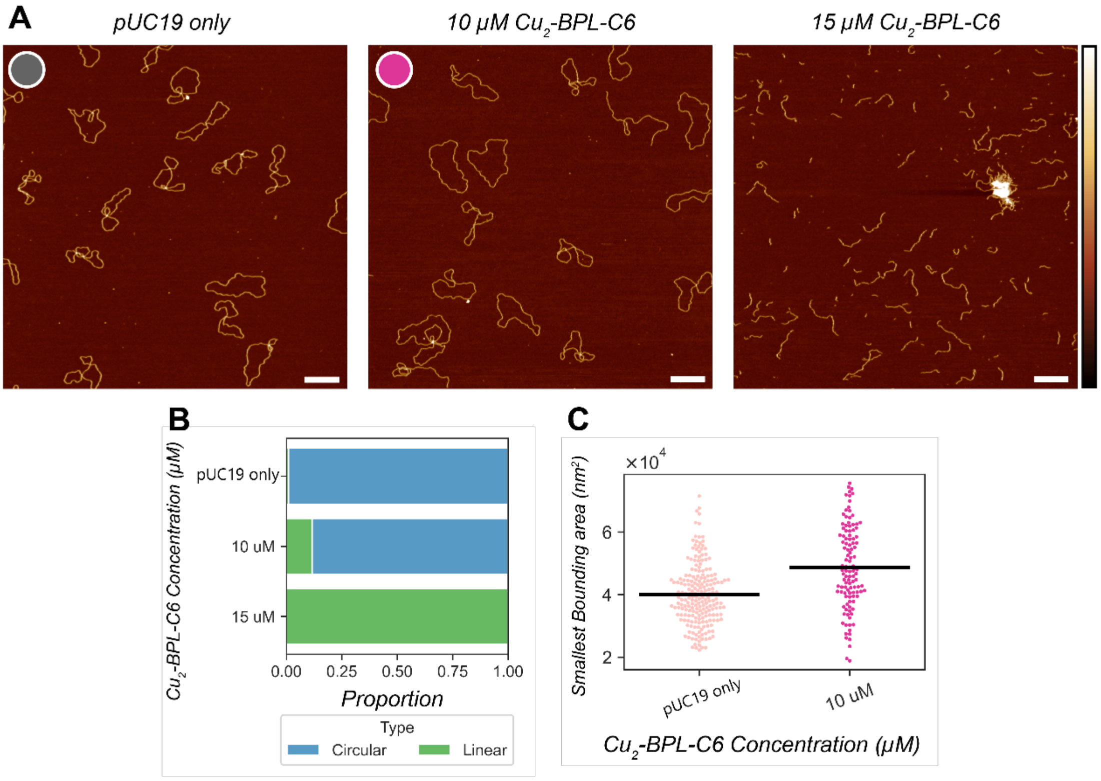

Figure S30: **A** In-liquid AFM showing the activity of Cu_2-BPL-C6 on pUC19 in the presence of 1 mM L-ascorbate for 30 min. Scale bars = 200 nm, height scales = -3 to 4 nm. **A**. Representative AFM images of untreated pUC19, 10 µM Cu_2-BPL-C6 treated pUC19 and 15 µM Cu_2-BPL-C6 treated pUC19. **B**. Quantification of the proportion of circular and linear molecules present in the images. **C**. Quantitative analysis of the smallest bounding area of individual circular molecules. N-values are as follows, pUC19 only: 213, 10 µM: 138, 15 µM: 381.

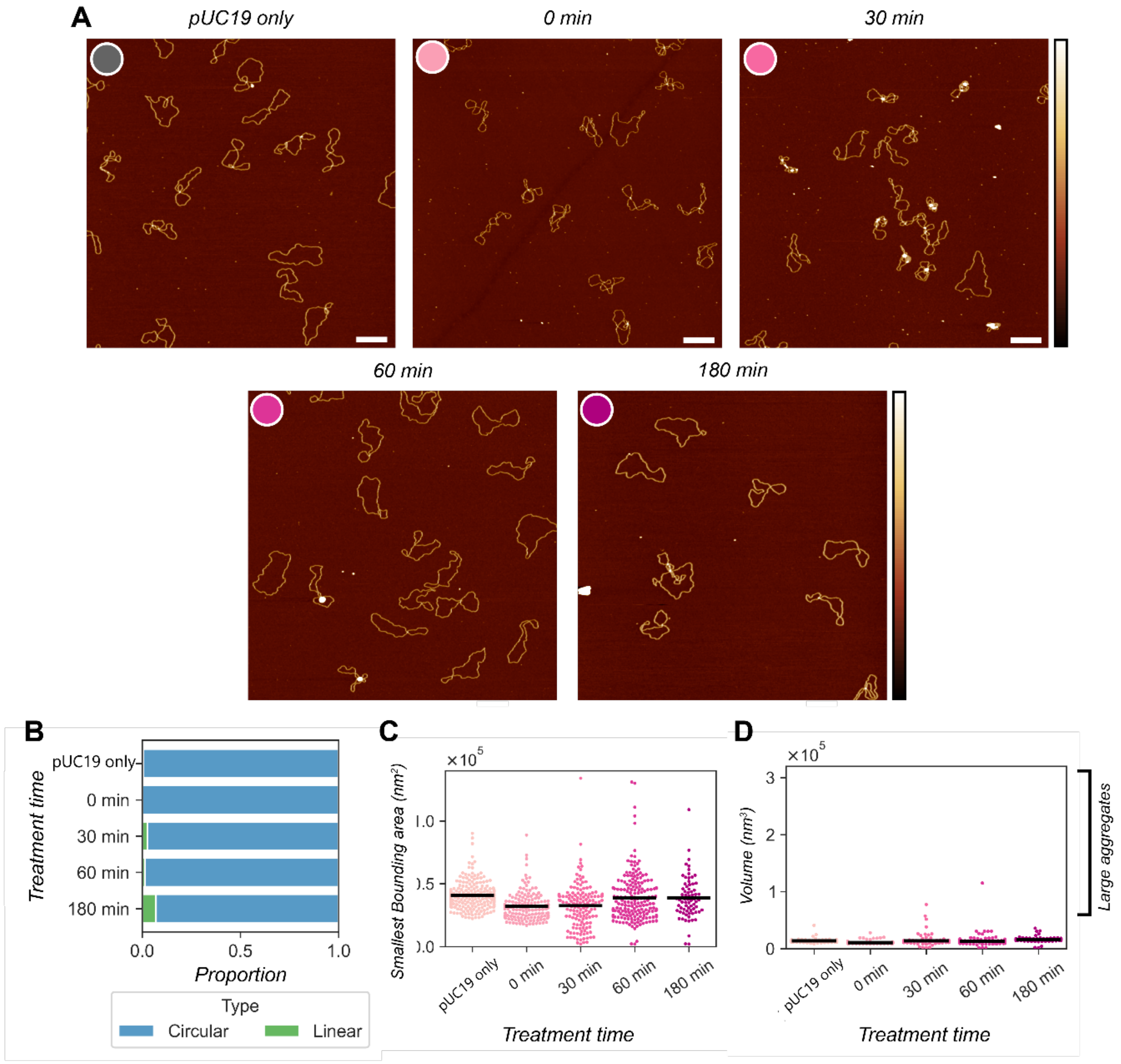

Figure S31: In-liquid AFM showing the activity of 20 µM Cu-Prodigiosin on pUC19. **A**. Representative AFM images of untreated pUC19, Cu-Prodigiosin treated pUC19 after 0, 30, 60, and 180 min. Scale bars = 200 nm, height scales = -3 to 4 nm. **B**. Quantification of the proportion of circular and linear molecules present in the images. **C, D**. Quantitative analysis of the smallest bounding area of individual circular molecules and the total volume of the masked grains. N-values are as follows - pUC19 only: 213, 0 min: 152, 30 min: 146, 60 min: 194, 180 min: 71.

References

1. Rigaku Oxford Diffraction, (2022), CrysAlisPro Software system, version 1.171.42.49, Rigaku Corporation, Oxford, UK.
2. C.B. Hübschle, G.M. Sheldrick and B. Dittrich. J. Appl. Cryst., 2011, **44**, 1281-1284.
3. O.V. Dolomanov, L.J. Bourhis, R.J. Gildea, J.A.K. Howard & H. Puschmann. J. Appl. Cryst., 2009, **42**, 339-341.
4. G.M. Sheldrick, Acta Cryst., 2015, **A71**, 3-8.
5. G.M. Sheldrick, Acta Cryst., 2015, **C71**, 3-8.
